# Interface Effects in Metal‐2D TMDs Systems: Advancing the Design and Development Electrocatalysts

**DOI:** 10.1002/advs.202500226

**Published:** 2025-03-26

**Authors:** Hao Hu, Zhongyuan Wang, Meilan Pan, Yumin Chen, Yinxi Han, Jiade Wang

**Affiliations:** ^1^ College of Environment Zhejiang University of Technology Hangzhou 310012 P. R. China; ^2^ Key Laboratory of Microbial Technology for Industrial Pollution Control of Zhejiang Province Hangzhou Hangzhou Zhejiang 310012 P. R. China

**Keywords:** electrocatalysis, interfaces bonded, interfaces doping/intercalation, metal‐2D TMDS interface structure, ohmic contact

## Abstract

2D transition metal dichalcogenides (2D TMDs) have emerged as promising candidates in electrocatalysis due to their unique band structures and tunable electronic properties. Nevertheless, establishing robust, low‐resistance contacts between TMDs layers and conductive supports has remained a challenge. Their atomically thin nature makes these layers prone to structural disruption and undesired chemical interactions, hampering charge transfer and diminishing catalytic efficiency. Recently, the visualization of microscopic interface behaviors and atomic layer interactions between metals and 2D TMDs has led to the introduction of ohmic contact metal‐TMDs electrocatalysts to address these challenges. Specifically, synergy at the metal‐2D TMDs interface endows the catalyst with new functionalities, including enhanced redox activity and selective reactant immobilization, thus helping address core challenges in energy conversion and storage. This work first examines the fundamental structural traits of 2D TMDs and introduces design principles and strategies for ohmic metal‐TMDs composites in electrocatalysis. The discussion covers methods for adjusting work function differences, constructing edge contacts in TMDs, incorporating interface doping/insertion, and engineering orbital hybridization or bonding interfaces. Additionally, this work analyzes the advantages, limitations, and future prospects of each approach, offering valuable insights for the development of efficient metal‐semiconductor catalysts, electrodes, and energy conversion and storage devices.

## Graphical abstract

This review examines the role of metal‐2D TMDs interfaces in electrocatalysis, emphasizing design principles and strategies for achieving ohmic contact catalysts. It provides valuable insights into improving energy conversion and storage technologies, highlighting the potential of metal‐2D TMDs composites for enhanced catalytic performance and stability.

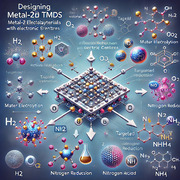

## 1 Introduction

Electrocatalysis, defined as the process of accelerating electrochemical reactions through electrocatalysts, was first introduced by Grubb in 1963.^[^ ^1^, ^2^ ^]^ Positioned at the intersection of electrochemistry and catalysis, electrocatalysis has found extensive application in fields including the controlled synthesis and functional assembly of nanomaterials,^[^ ^3^, ^4^, ^5^ ^]^ electrochemical energy conversion,^[^ ^6^, ^7^, ^8^, ^9^, ^10^ ^]^ electrochemical sensors and biosensors,^[^ ^11^, ^12^, ^13^, ^14^ ^]^ detection and analysis,^[^ ^15^, ^16^, ^17^, ^18^ ^]^ as well as the degradation of organic waste and wastewater treatment.^[^ ^12^, ^13^, ^14^ ^]^ Particularly, the urgent demand for clean and sustainable energy has led to the rapid advancement of electrocatalysis in fuel cells, water splitting electrolyzers, carbon dioxide emission reduction (CO_2_RR) and etc.^[^ ^16^, ^17^ ^]^ Scientific opportunities and challenges thus come out, for example, the slow kinetics of the four‐electron transfer processes in oxygen evolution reaction (OER) and oxygen reduction reaction (ORR);^[^ ^18^, ^19^ ^]^ the limited kinetics of hydrogen evolution reaction (HER) and hydrogen oxidation reaction (HOR) in alkaline electrolyte (being much slower in alkaline environments compared to acidic ones);^[^ ^20^, ^21^ ^]^ and low current density in carbon dioxide emission reduction and nitrogen reduction reaction (NRR).^[^ ^22^, ^23^ ^]^ The key to solving these problems lies in the design and development of advanced electrocatalysts, which determine the activity, selectivity and durability of the electrocatalytic system. According to the Sabatier principle, an ideal electrocatalyst should maintain a moderate binding strength with the reactants: it can effectively adsorb the reactants; and rapid release the products.^[^ ^24^ ^]^ On this basis, the interaction between the active layer and the support provides a new perspective for the development of efficient catalysts—rationally apply the support effect to modulate electrocatalytic activity and reduce the cost.

2D transition metal dichalcogenides (2D TMDs) exhibit high energy conversion efficiency and distinctive layered architectures, characterized by robust intralayer covalent bonds and interlayer van der Waals interactions.^[^ ^25^, ^26^, ^27^ ^]^ These features link layered‐structure control with electrocatalytic performance,^[^ ^28^ ^]^ especially through effective interfacial synergy between 2D TMDs and their supports, which promotes electron transport, relieves interlayer stress, and improves mechanical durability.^[^ ^29^, ^30^, ^31^, ^32^ ^]^ However, establishing high‐quality interfacial contacts between 2D TMDs and supports remains challenging due to their atomic‐scale thinness and fragile lattices.^[^ ^33^, ^34^, ^35^ ^]^ On one hand, conventional deposition methods may induce atomic disorder on 2D TMDs surfaces,^[^ ^36^, ^37^, ^38^ ^]^ causing severe fermi level pinning (FLP) effects and impeding electron transport.^[^ ^39^, ^40^, ^41^ ^]^ On the other hand, lattice mismatches at the contact interface can lead to lattice distortions, stress accumulation, and misalignment of energy levels,^[^ ^42^, ^43^, ^44^ ^]^ ultimately forming interface barriers and undermining the stability of electron transmission channels.

By optimizing the interface structure between metals and 2D TMDs, the construction of ohmic contact‐based composite electrocatalysts, with negligible interfacial resistance, represents a promising strategy. The electron transport barrier caused by schottky junction and the charge distribution inhomogeneity caused by local electric field effect can be alleviated by optimizing the contact mode or microstructure between the support and 2D TMDs.^[^ ^45^ ^]^ This ensures unhindered electron transfer across the interface, significantly reducing energy losses during charge transport.^[^ ^46^ ^]^ For example, Kang et al. conducted a systematic density functional theory (DFT) study on the contacts between single‐layer TMDs and various metal substrates, initiating vigorous discussions on the impacts of orbital overlap, schottky barriers, and tunnel barriers on interface contact effects.^[^ ^47^ ^]^ Charlie et al. studied the influence of different supports on the hydrogen adsorption free energies at MoS_2_ edges. It was revealed that the physical separation (𝑑) at edge contacts is much smaller than at top contacts, due to atomic orbital overlap between the support and TMDs, which reduces the height of tunnel barriers and schottky barriers, thereby optimizing the electron transport between the support and TMDs edge sites.^[^ ^48^ ^]^ More strategies have been proposed to optimize the contact structure between supports and 2D TMDs, such as low work‐function metal contacts,^[^ ^49^ ^]^ tuning the geometry or crystal phase of TMDs,^[^ ^50^, ^51^ ^]^ inserting buffer layers,^[^ ^52^ ^]^ and employing ultra‐high vacuum evaporation.^[^ ^53^ ^]^ These approaches aim to reduce interfacial contact resistance, accelerate charge transfer, and lower reaction energy barriers. Furthermore, contact interfaces can be reinforced through orbital overlap or hybridization, resulting in robust electrocatalytic performance.

In this article, we begin by discussing the structural features of TMDs and the fundamental principles of metal‐TMDs contacts. We explore the interface structure and properties between metals and 2D TMDs, analyzing the advantages and drawbacks of van der Waals and non‐van der Waals contacts. The article further addresses the relationship between interface effects (such as tunneling barriers, schottky barriers, and fermi‐level pinning) and the contact resistance at the interface. We then elaborate on the current strategies for controlling interface structures and their impact on the contact resistance between metals and 2D TMDs, with a particular focus on achieving ohmic contact and its applications in electrochemistry. Finally, we identify key areas that require further exploration in the metal‐TMDs contact process and offer insights on these aspects.

## 2 Fundamentals of 2D TMDs

As an emerging material class in the post‐graphene era, the chemical versatility of 2D TMDs spans a wide range of material types, including true metals (e.g., NbS_2_ and VSe_2_), semimetals (e.g., WTe_2_ and TiSe_2_), semiconductors (e.g., MoS_2_ and WS_2_), and insulators (e.g., HfS_2_).^[^ ^54^ ^]^ Unlike other 2D materials such as graphene, black phosphorus, hexagonal boron nitride, and transition metal phosphides or carbides, TMDs are distinguished by their unique crystal phases and tunable band gaps. For example, the 2H phase exhibits semiconductor‐like characteristics, the 1T phase is metallic, and the 1T' phase is semimetallic.^[^ ^55^, ^56^ ^]^ Furthermore, monolayer TMDs display a direct band gap, while multilayer TMDs exhibit an indirect band gap due to interlayer interactions.^[^ ^51^ ^]^ These characteristics allow the phase and band gap of TMDs to be tailored for specific applications, enabling customization of their properties to meet the unique requirements of interactions with different metal substrates and electrochemical needs.

### 2.1 Composition and Crystal Stacking of 2D TMDs

**Figure 1:**
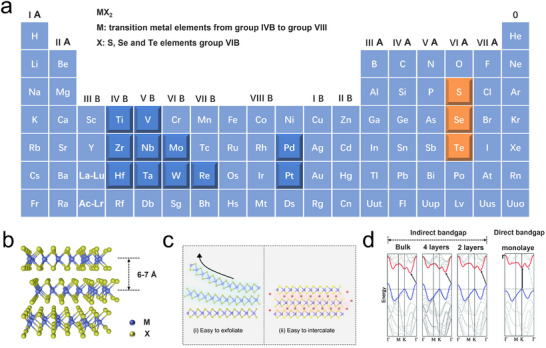
a) Composition of TMDs. b) Schematic of 2D MX_2_. M denotes a transition metal and X denotes a chalcogen. c) Schematic illustration of exfoliation and intercalation of 2D TMDs materials.^[^ ^58^ ^]^ Copyright 2024, American Chemical Society. d) Calculated band structures of bulk MoS_2_, and MoS_2_ flakes with four layers, two layers, and one layer. The arrows in each part indicate the lowest energy transitions.^[^ ^61^ ^]^ Copyright 2023, Wiley‐VCH.

As depicted in **Figure** **1**a, TMDs are commonly represented by the formula MX_2_, where M denotes transition metal elements from groups IVB to VIIIB, and X refers to chalcogen elements from group VIA, including sulfur (S), selenium (Se), and tellurium (Te).^[^ ^54^ ^]^ TMDs are quintessential 2D layered nanomaterials, characterized by unique structural and electronic properties that are highly sensitive to dimensionality. Each monolayer of TMDs consists of three atomic layers: two hexagonal planes of chalcogen atoms are sandwiched between a metal atom layer, forming an “X–M–X” sandwich structure.^[^ ^57^ ^]^ The transition metal atoms are covalently bonded to the chalcogen atoms via strong ionic‐covalent interactions, while adjacent layers are held together by weaker van der Waals (vdW) forces, with interlayer spacing typically ranging from 6 to 7 Å (Figure 1b).^[^ ^54^ ^]^ This relatively weak interlayer bonding enables TMDs to be exfoliated into monolayers or few‐layer flakes, leading to significant alterations in their properties compared to bulk materials (Figure 1c).^[^ ^58^ ^]^ For instance, while the bulk form of many TMDs exhibits an indirect bandgap, the reduction in dimensionality to monolayer results in the emergence of a direct bandgap. Taking 2H‐MoS_2_ as an example, when the material's thickness is reduced to a monolayer, its electronic bandgap increases from 0.88 eV in the bulk form to 1.71 eV in the monolayer form, transitioning from an indirect bandgap semiconductor to a high‐electron‐mobility direct bandgap material (Figure 1d).^[^ ^59^, ^60^, ^61^ ^]^ This dramatic change in electronic properties endows TMDs with remarkable potential for a wide range of applications, particularly in fields such as optoelectronics, photovoltaics, and electrocatalysis, where their inherent 2D characteristics can substantially enhance performance and efficiency. The ability to engineer and tune the electronic properties of TMDs through dimensional control has sparked considerable interest in their potential to address key challenges in energy conversion, catalysis, and sensor technologies. Their scalability and tunability, coupled with a high surface area and exceptional conductivity, make TMDs ideal candidates for advanced applications, offering significant improvements over traditional bulk materials. These capabilities position TMDs as an emerging class of materials for the next generation of high‐performance, scalable devices.

### 2.2 Correlation between Crystal Phases and Electronic Band Structure of 2D TMDs

**Figure 2:**
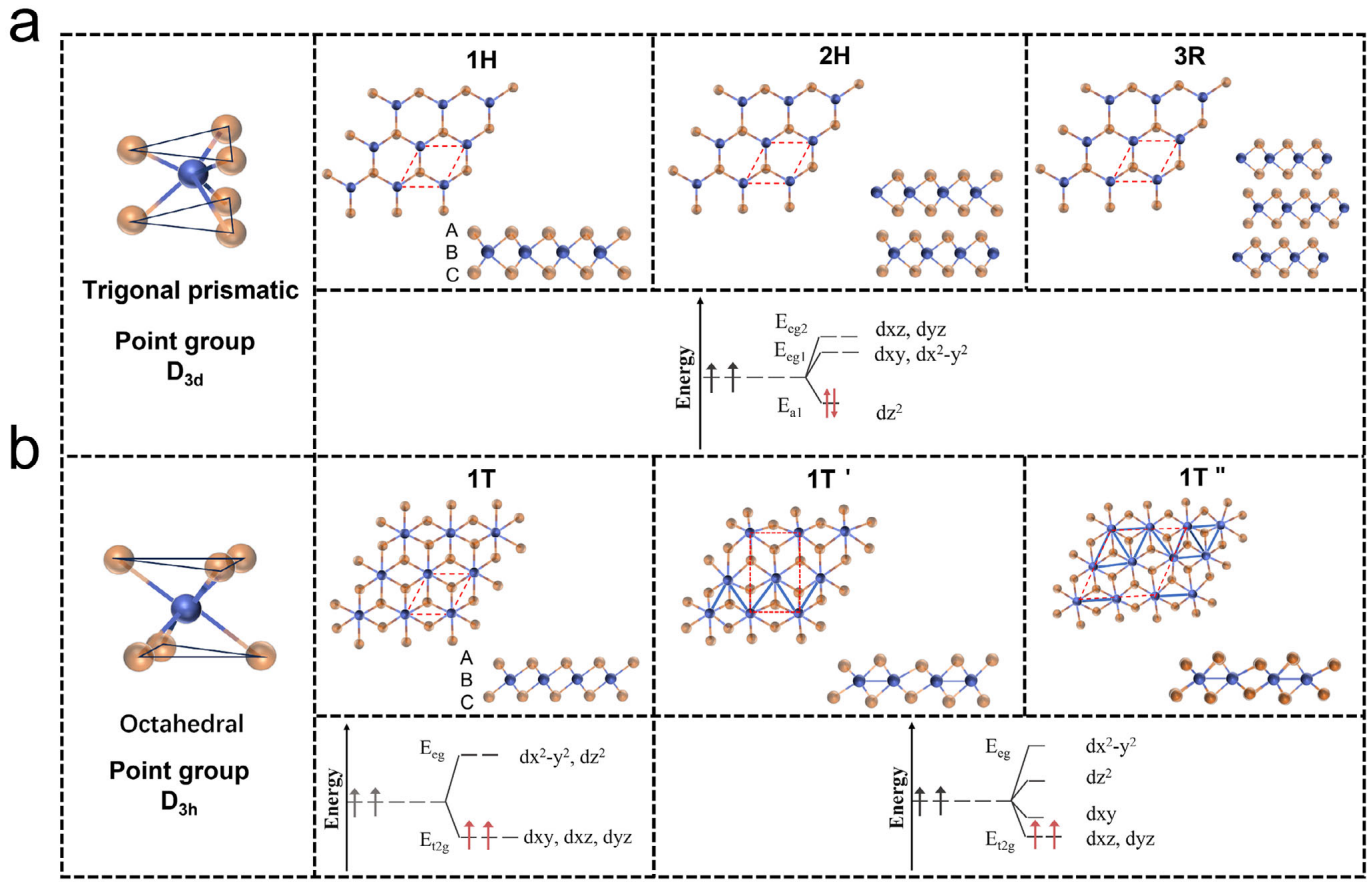
Schematic diagram of TMDs, which have various polycrystalline phases and corresponding d orbital fillings according to the coordination structure and stacking order, including 1H, 2H, 3R, 1T, 1T′, and T″ phases. a) Triangular prism structure. b) Octahedral structure.

TMDs are known for exhibiting a wide array of crystal phases, including 1T, 1T′, T″, 2H, and 3R.^[^ ^62^ ^]^ These materials adopt two primary coordination modes: trigonal prismatic and octahedral coordination, which are determined by variations in the outermost electron count, particularly the filling of the d orbitals. As depicted in **Figure** **2**, the 1T, 1T′, and T″ phases exhibit octahedral coordination, while the 2H and 3R phases are characterized by trigonal prismatic coordination.^[^ ^54^ ^]^ The structural characteristics of these phases can be summarized as follows: T‐phase TMDs feature an atomic stacking sequence of A–B–C (X–M–X′), while the 1T′ and 1T″ phases exhibit monoclinic and orthorhombic symmetries, respectively, due to distortions caused by the dimerization of transition metal atoms in the 1T phase. In contrast, the 2H and 3R phases adopt an A–B–A (X–M–X) stacking arrangement, exhibiting hexagonal close‐packed symmetry across multiple layers.^[^ ^63^ ^]^

According to ligand field theory, the non‐bonding d bands in TMDs lie within the gap between the bonding (σ) and anti‐bonding (σ^*^) bands that separate the M–X bonds, whether the material is in the 1H or 1T phase.^[^ ^54^ ^]^ In this context, the d orbitals of transition metal centers in octahedral and trigonal prismatic coordination (D*_3h_* and D*_3d_* symmetries, respectively) split into distinct sets. In octahedral coordination, the orbitals split into $d_{x^{2}-y^{2}},d_{z^{2}}$ (*E_eg_*) and *d_yz_*, *d_xz_*, *d_xy_* (*E_t2g_*) orbitals, while in trigonal prismatic coordination, the orbitals split into $d_{z^{2}}$ (*E_a1_*), $d_{x^{2}-y^{2}}$, *d_xy_* (*E_eg1_*), and *d_xz_*, *d_yz_* (*E_eg2_*) orbitals.^[^ ^64^, ^65^, ^66^ ^]^ In thermodynamically stable 2H TMDs (Group VI), the *E_a1_* orbital is fully occupied, while the *E_eg1_ and E_eg2_* orbitals remain unfilled, giving the material its semiconductor behavior (Figure 2a). In contrast, octahedrally coordinated 1T‐phase TMDs feature partially filled *E_t2g_* bands, which accounts for their metallic properties (Figure 2b).^[^ ^67^ ^]^ This pronounced sensitivity of TMDs' electronic behavior to their structural phase highlights their potential for directed modulation, particularly in electrocatalysis. This aspect has become a central focus of current research, with work by Zhou et al. offering a detailed review of design strategies and optimization principles for unsaturated electronic structures in 2D TMDs. Their study focuses on the electronic structural behavior and modulation rules of MoS_2_ in various crystal phases to optimize its performance in electrocatalytic hydrogen evolution.^[^ ^68^ ^]^ For example, phase transitions can be strategically controlled by donating additional electrons to the *E_eg_* band of the trigonal prismatic 2H phase, destabilizing its lattice and promoting its transformation to the metallic 1T phase.^[^ ^69^, ^70^ ^]^ Conversely, applying external strain such as compression or stretching to the octahedrally coordinated 1T phase can induce a spontaneous transformation to the 1T' phase, lowering its energy and improving stability via Jahn‐Teller distortion effects.^[^ ^71^, ^72^ ^]^ These findings underscore the remarkable versatility of TMDs in their chemical composition, structural symmetry, and electronic properties, making them highly attractive for applications in energy conversion, storage, and electrocatalysis. Given their tunable characteristics, TMDs represent a promising class of materials for future high‐performance devices.

### 2.3 Metal‐2D TMDs Synthesis Strategies: From Exfoliation to In Situ Growth

The selection of substrate is a crucial factor that determines the efficiency and performance of electrocatalytic systems, as it directly influences the electronic interactions at the support‐active site interface.^[^ ^73^ ^]^ Conductive substrates, such as graphite, glassy carbon (GC), highly ordered pyrolytic graphite (HOPG), and carbon cloth (CC), are commonly used as supports for electrocatalysts, providing the necessary electrical conductivity to facilitate effective charge transfer and promote catalytic processes.^[^ ^74^ ^]^ However, materials like graphite, GC, and CC typically exhibit weak interface interactions with the catalyst, which can hinder efficient electron transfer from the electrode to the active sites.^[^ ^45^ ^]^ In contrast, metal substrates offer superior performance, making them ideal platforms for electrocatalytic reactions: (1) their active d‐orbital electrons provide high compatibility for the nucleation and growth of TMDs, facilitating their direct integration into electrochemical systems; (2) their excellent conductivity ensures optimal charge transfer at the interface; (3) strong interface coupling between MX_2_ (TMDs) and specific metals enhances electronic interactions at the electrode surface, leading to exceptional catalytic efficiency. Therefore, the choice of substrate material plays a decisive role in determining the overall performance of electrocatalysts.

#### 2.3.1 Exfoliation of TMDs and Their Integration with Metal Substrates

The van der Waals interactions between atomic layers of 2D TMDs make exfoliation one of the most widely used techniques for obtaining high‐quality monolayer or few‐layer TMDs, such as MoS_2_, WS_2_, and MoSe_2_.^[^ ^75^ ^]^ Common exfoliation methods include mechanical exfoliation (e.g., the scotch tape method),^[^ ^76^ ^]^ liquid‐phase ultrasonic exfoliation,^[^ ^57^ ^]^ and electrochemical intercalation exfoliation.^[^ ^77^ ^]^ By applying mechanical force to bulk crystals, these techniques efficiently isolate individual layers of TMDs while preserving their intrinsic chemical structure.^[^ ^78^ ^]^ This process enables the transformation of the indirect bandgap characteristic of bulk TMDs into a direct bandgap with high charge carrier mobility, a key feature of monolayers.^[^ ^79^ ^]^

**Figure 3:**
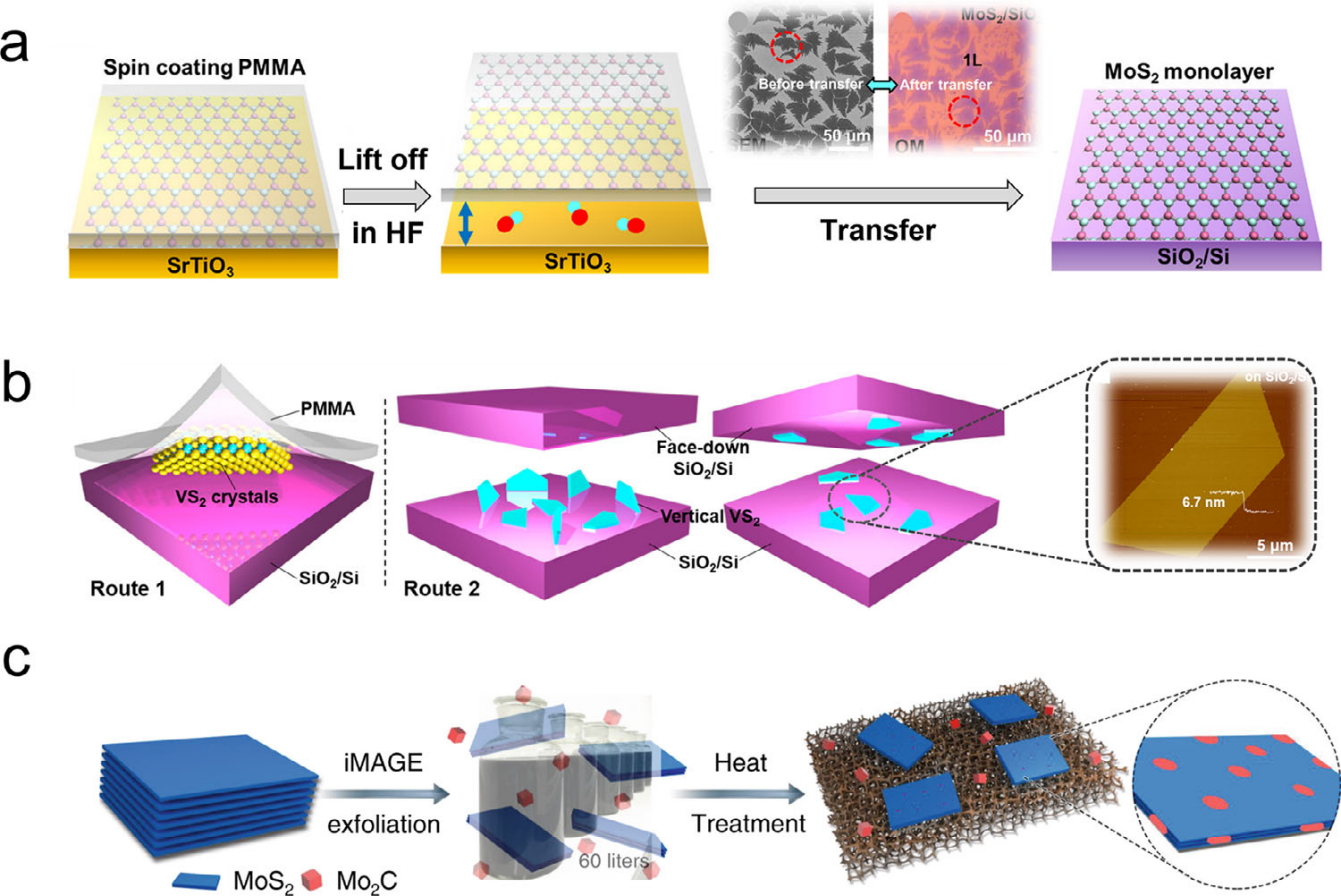
a) Schematic diagram of the process of perfect transfer of quasi‐dendritic MoS_2_ flakes to SiO_2_/Si, (Inset: SEM image and optical image of single‐layer MoS_2_ flakes).^[^ ^81^ ^]^ Copyright 2014, American Chemical Society. b) Schematic illustration of the two typical transfer methods for the VS_2_ crystals.^[^ ^82^ ^]^ (Illustration: AFM height image of a VS_2_ nanosheet.) Copyright 2017, American Chemical Society. c) Schematic of the fabrication method of MoS_2_‐based catalysts.^[^ ^83^ ^]^ Copyright 2020, Springer Nature.

Exfoliated TMDs can then be integrated with metal substrates (e.g., Au, Pt, Cu) through techniques such as drop‐casting, spin‐coating, or electrochemical deposition to meet the requirements of electrochemical applications.^[^ ^80^ ^]^ For example, Zhang and colleagues developed a PMMA‐assisted transfer method for effectively transferring monolayer MoS_2_ onto Au foils. (**Figure** **3**a) This process involves an etching step that detaches the native MX_2_ (supported by the polymer film) from the growth substrate. The transferred MoS_2_ flakes, exhibiting a branched morphology, maintain their structural integrity and high crystal quality. This method ensures the successful transfer of high‐quality MoS_2_ without compromising its crystalline properties.^[^ ^81^ ^]^ In another example, Ji and colleagues employed a polymer‐free transfer method to fabricate VS_2_ nanosheets, (Figure 3b) which exhibited a clean adsorbed layer‐substrate interface and exceptional electrical conductivity (≈3 × 10^3^ S cm⁻^1^).^[^ ^82^ ^]^ Furthermore, the development of solution‐processable 2D‐TMDs inks has introduced a novel strategy for fabricating metal‐TMDs composites. Liu et al. employed a Mo_2_C‐assisted intermediate grinding exfoliation (iMAGE) technique to exfoliate MoS_2_ from bulk crystals and disperse it in water. They then utilized dip‐coating to load the material onto high‐surface‐area Cu foam, resulting in a highly efficient catalyst for the HER (Figure 3c).^[^ ^83^ ^]^ However, despite the advancements in exfoliation and functionalization methods to improve the interaction between TMDs and metal surfaces, several challenges remain.^[^ ^84^ ^]^ The weak van der Waals interactions at the interface result in high contact resistance, and the low yield and random size distribution of exfoliated flakes limit the scalability and electrochemical stability of these systems.^[^ ^51^ ^]^ These factors present significant barriers to the widespread application of metal‐TMDs hybrids in electrocatalysis and other electrochemical processes.

#### 2.3.2 Deposition of TMDs on Metal Substrates

**Figure 4:**
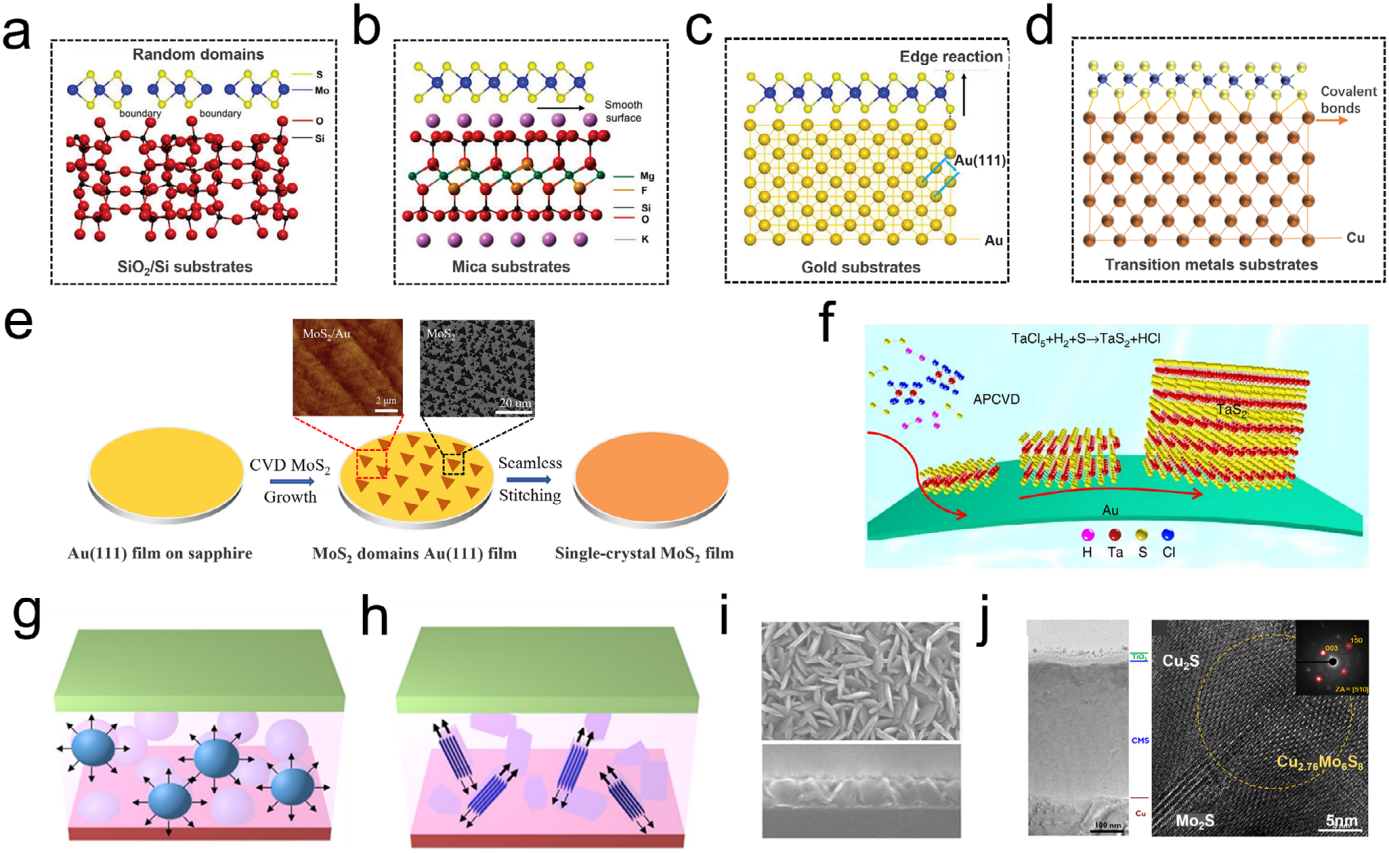
The interactions of 2D TMDs with commonly used substrates. a) SiO_2_/Si; b) Mica; c) Nobel metal (Au); d) Transition Metals (Cu).^[^ ^87^ ^]^ Copyright 2023, Wiley‐VCH. e) Schematic diagram of the growth of single crystal MoS_2_ on Au. (Inset: SEM image and AFM morphology image of MoS_2_ domains growth on the Au (111) substrates.)^[^ ^93^ ^]^ Copyright 2021, Wiley‐VCH. f) Schematic illustration of the APCVD growth process.^[^ ^94^ ^]^ Copyright 2017, Springer Nature. g) Illustration of a typical bulk heterojunction (BHJ). h) Illustration of the anisotropic charge transport in bulk. i) SEM cross‐sectional image of CMS. j) TEM image of TiO_2_/CMS/Cu and high‐resolution TEM (HR‐TEM) image of Cu_2.76_Mo_6_S_8_ Chevrel phase formed at the interface of MoS_2_ and Cu_2_S.^[^ ^95^ ^]^ Copyright 2017, American Association for the Advancement of Science.

In the deposition of TMDs, the selection of the substrate plays a crucial role in anchoring the source material, facilitating nucleation, and driving epitaxial growth.^[^ ^85^, ^86^ ^]^ The substrate significantly influences the final product's thickness, microstructure, and crystallinity, including nucleation density, nucleation size, and epitaxial orientation.^[^ ^87^ ^]^ These factors are essential for producing 2D TMDs with the desired morphology and dimensions. As depicted in **Figure** **4**a,b, among various substrates, materials lacking dangling bonds, such as SiO_2_/Si and mica, promote lateral atomic diffusion, which in turn hinders stable growth.^[^ ^88^, ^89^, ^90^ ^]^ In contrast, metal substrates with high intrinsic electron mobility and lattice symmetries that closely match those of TMDs (e.g., Au, Cu, Ni) facilitate much stronger chemical interactions between the edge atoms of TMDs and the metal substrate, far exceeding the intralayer van der Waals forces.^[^ ^91^, ^92^ ^]^ As a result, nucleation is predominantly governed by edge interactions, promoting crystal nucleation (Figure 4c,d).

Notably, Au substrates exhibit remarkable chemical inertness toward VI‐A precursor elements, making them an ideal platform for the growth of large‐area 2D TMDs.^[^ ^93^ ^]^ For instance, Li et al. demonstrated the deposition of Au (111) onto c‐plane sapphire to create a single‐crystal Au (111) thin film substrate. As shown in Figure 4e, MoS_2_ grown on the Au (111) substrate forms seamlessly, yielding highly oriented, wafer‐scale MoS_2_ films. By controlling the temperature gradient, MoS_2_ is rotated along the optimal growth direction, achieving over 99% rotational alignment, with an average flake size of ≈4.1 µm.^[^ ^87^ ^]^ Similarly, Shi et al. directly synthesized uniform, thickness‐tunable monolayer 2H‐TaS_2_ films on Au substrates using a simple chemical vapor deposition (CVD) process (Figure 4f).^[^ ^94^ ^]^ In contrast to gold, transition metals such as Cu and Ni, which possess more active d‐orbital electrons, exhibit strong electron‐donating or accepting behavior at their surfaces. During deposition, these metals form stable covalent bonds with sulfur atoms in TMDs. For example, Shin et al. grew MoS_2_ on self‐supporting Cu foil via CVD, where MoS_2_ nanosheets were embedded within a Cu_2_S matrix, forming a bulk Cu‐Mo‐S heterostructure (BLHJ). This strategy has been extended to other transition metals, such as Ni (Figure 4g–j).^[^ ^95^ ^]^ However, in many deposition processes, lattice mismatch between the metal substrate and TMDs induces significant strain at the deposition interface, often leading to cracks or delamination in the TMDs films. This challenge severely limits their scalability and large‐scale applications, particularly in catalytic electrode systems.^[^ ^92^, ^96^ ^]^

#### 2.3.3 In Situ Growth of TMDs on Homologous Metal Substrates

In traditional metal‐2D TMDs systems (e.g., MoS_2_/Au, WS_2_/Pt), lattice mismatch and weak interfacial bonding significantly hinder charge transport efficiency and device stability. For instance, in the MoS_2_/Au heterojunction, the lattice constant of MoS_2_ (3.16 Å) differs substantially from that of Au (2.88 Å), resulting in a mismatch of ≈9.7%. This lattice mismatch induces the formation of dislocations and dangling bonds at the interface,^[^ ^91^, ^97^ ^]^ which lead to the creation of a schottky barrier (≈0.3–0.5 eV), obstructing electron transport across the interface and resulting in an interface resistance > 50 Ω cm⁻^2^.^[^ ^98^ ^]^ Furthermore, conventional contact interfaces typically rely on physical adsorption or van der Waals interactions, which yield low interface binding energies (<0.1 eV atom⁻^1^), making them vulnerable to significant volume changes and bubble formation under high potential conditions.^[^ ^99^ ^]^

**Figure 5:**
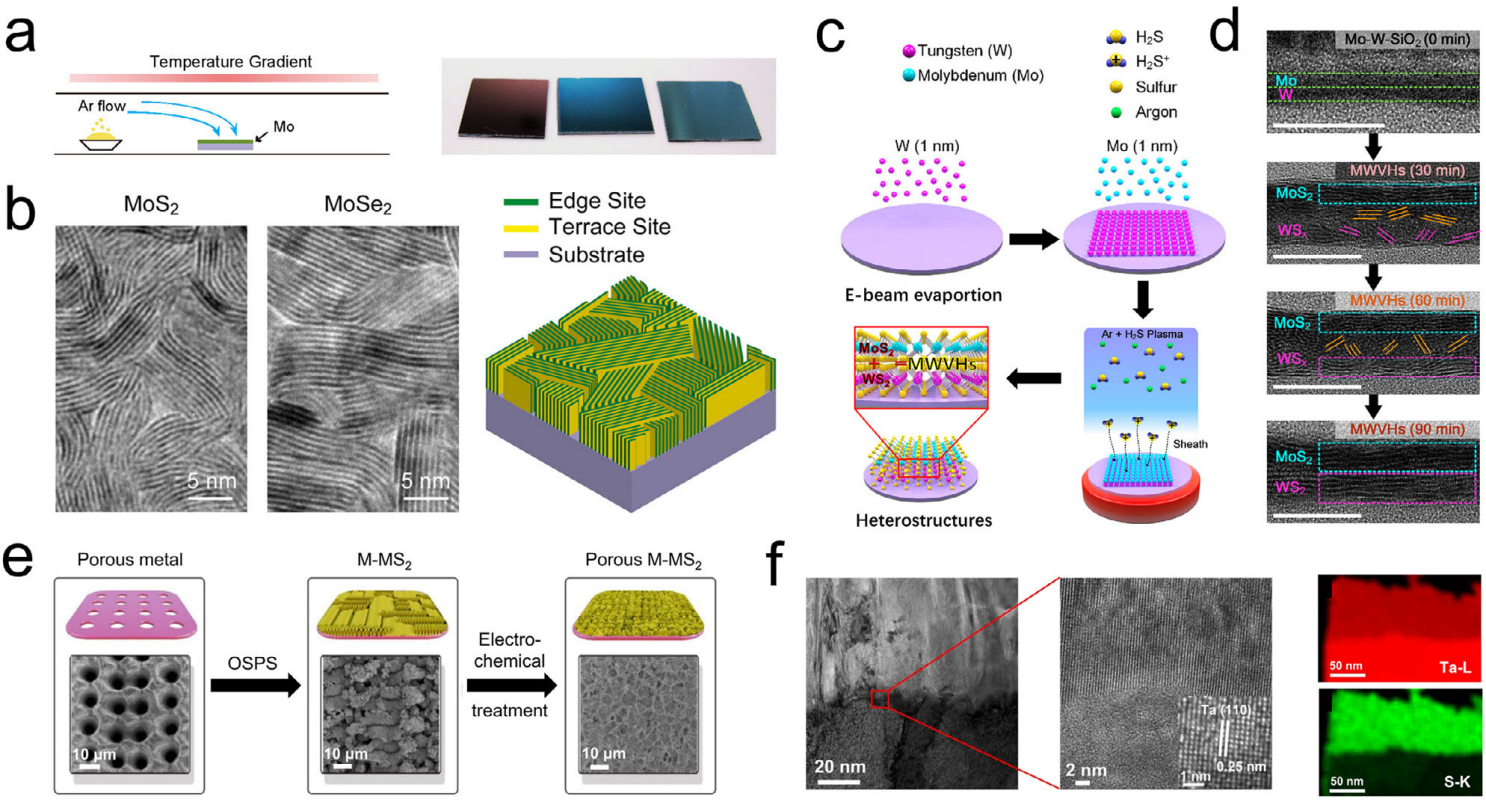
a) Schematic of the synthesis setup in a horizontal tube furnace (left). Digital photos of a pristine oxidized silicon (300 nm SiO_2_/Si) substrate, a MoS_2_ film on oxidized silicon, and a MoSe_2_ film on oxidized silicon (right). b) Structure of edge‐terminated molybdenum chalcogenide films with the layers aligned perpendicular to the substrate, maximally exposing the edges of the layers.^[^ ^102^ ^]^ Copyright 2013, American Chemical Society. c) Schematic of the synthesis of MoS_2_–WS_2_ vertical heterostructures (MWVHs) via single‐step penetrative sulfurization by PE‐CVD at 300 °C. d) Illustration of the mechanism using time‐dependent Ar + H_2_S plasma and cross‐sectional HR‐TEM images (scale bar: 10 nm).^[^ ^104^ ^]^ Copyright 2021, American Chemical Society. e) The OSPS synthesis process of Ta‐TaS_2_ MC and corresponding SEM images. f) TEM cross‐sectional image of Ta‐TaS_2_ MC and magnified image of the interface and corresponding STEM‐EDS elemental map.^[^ ^103^ ^]^ Copyright 2021, Springer Nature.

To overcome these challenges, researchers have increasingly focused on innovative strategies for the in situ growth of TMDs on isostructural metal substrates (e.g., Mo foil for MoS_2_ growth, W foil for WS_2_ growth).^[^ ^100^, ^101^ ^]^ The key to this approach lies in the isostructural compatibility between the metal substrate and the transition metal elements in the TMDs, ensuring chemical consistency. Through thermodynamically driven sulfurization or selenization reactions, this strategy facilitates atomic‐level epitaxial growth and covalent bonding at the metal‐TMDs interface. For example, Cui et al. used Mo foil as the Mo source and employed a rapid sulfurization/selenization process via chemical vapor deposition (CVD) to fabricate integrated Mo/MoS_2_ and Mo/MoSe_2_ electrodes. As illustrated in **Figure** **5**a, during high‐temperature sulfurization/selenization, the anisotropic interlayer structure of MoS_2_/MoSe_2_ allows sulfur/selenium vapors to diffuse more efficiently along the van der Waals gaps between the layers than across the layers, promoting vertical growth of MoS_2_/MoSe_2_ on the Mo foil (Figure 5b).^[^ ^102^ ^]^ Similarly, Liu et al. developed an in situ solid‐phase oriented synthesis (OSPS) method to fabricate monolithic catalysts (Figure 5e). After laser etching the Ta (Mo, Nb) substrate, it was pre‐oxidized to form Ta_2_O_5_ (MoO_3_, Nb_2_O_5_), which was subsequently sulfurized along the oxidation path (Ta_2_O_5_ → TaS_2_), resulting in the formation of a highly ordered porous structure (Figure 5f).^[^ ^103^ ^]^ Using a different strategy, Seok et al. employed various techniques to synthesize wafer‐scale MoS_2_‐WS_2_ vertical heterostructures on substrates pretreated with plasma at 300 °C (Figure 5c). The synthesis mechanism of the heterostructure was determined using high‐resolution transmission electron microscopy (HRTEM) (Figure 5d). With the assistance of Ar plasma, H_2_S permeated through the underlying layer and, through ion bombardment, reacted with the Mo‐W layer deposited on the substrate, resulting in the direct formation of a uniform wafer‐scale MoS_2_‐WS_2_ vertical heterostructure.^[^ ^104^ ^]^ Unlike heteroepitaxy using non‐isostructural metals, the high lattice matching between TMDs and isostructural metal substrates enhances compatibility, promoting uniform nucleation and controlled growth. Moreover, the in situ visualization of the growth process opens exciting possibilities for developing scalable, high‐quality TMDs materials with tailored properties. For example, by controlling the deposition temperature of TMDs on homologous metal substrates, one can modulate their surface geometry, or introduce in situ doping of other elements during deposition to facilitate changes in the electronic structure of the TMDs surface. These strategies, along with the electronic interactions between TMDs and metal substrates, show unparalleled potential in energy storage and electrocatalysis, making them a key focus of our ongoing research (**Table** **1**).

**Table 1:** Characteristics of preparation methods for different metal‐2D TMDs systems.

Material	Preparation methods	Interface features	Limitations	Refs.
MoS_2_/Au	Exfoliation transfer	Van der Waals interface	The main limitations of the exfoliation‐transfer process include high contact resistance, low yield with uneven size distribution, and poor interface stability.	[81]
HC‐MoS_2_/Mo_2_C	Exfoliation transfer			[93]
2H‐TaS_2_/Au	Metal substrate deposition	Van der Waals interface/covalent bond interface	The lattice mismatch between the metal substrate and TMDs may induce significant strain at the interface, leading to the formation of cracks or delamination in the TMDs films.	[94]
CMS	Metal substrate deposition			[95]
Mo/MoSe_2_	In situ growth of homologous metals	Covalent bond interface	The limitations in substrate material selection, the complexity of growth process control, and issues related to material size and uniformity.	[102]
Ta/TaS_2_ MC	In situ growth of homologous metals			[103]

## 3 Metal‐2D TMDs Interface Characteristics

### 3.1 Interface Structure

**Figure 6:**
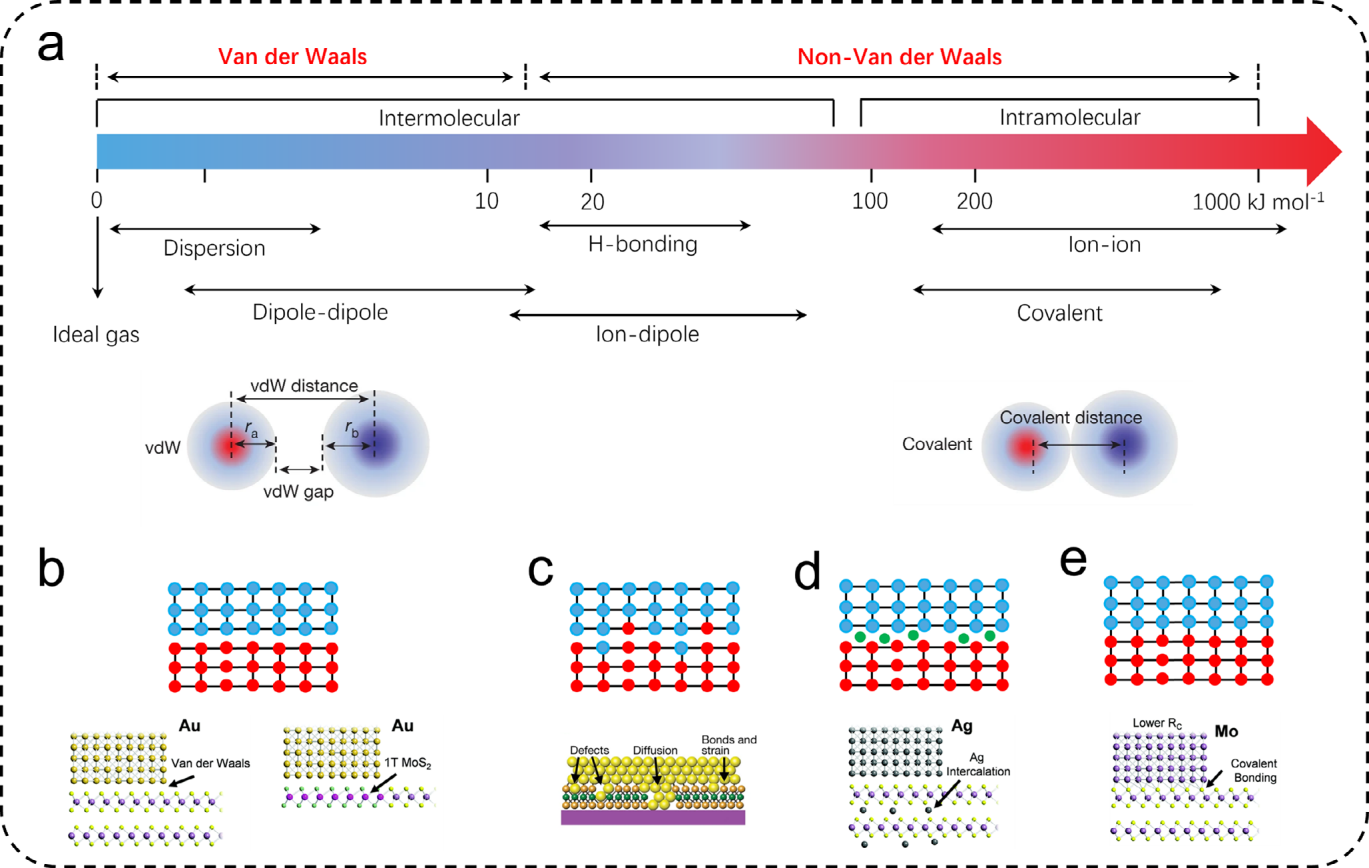
a) Energies of various molecular interactions and Schematic illustration of the vdW gap and the vdW distance in a covalent‐bonded system and a vdW system. vdW interaction is the weakest intermolecular interaction (also termed physical interaction), much smaller than typical intramolecular interactions (also termed chemical interactions).^[^ ^30^ ^]^ Copyright 2019, Springer Nature. b) Atomic structure at the van der Waals interface between metals and 2D TMDs. The atomic structure at the non‐van der Waals interface between metals and 2D TMDs: c) atomic disorder/defects at the interface in the unbonded state;^[^ ^111^ ^]^ Copyright 2018, Springer Nature. d) intercalation of heteroatoms at the interface in the unbonded state (Ag contact leads to Ag insertion between MoS_2_ layers); e) atomic interface in the bonded state (Mo contacts and forms covalent bonds with the underlying MoS_2_).^[^ ^34^ ^]^ Copyright 2018, Royal Society of Chemistry.

The interface structure between metals and 2D TMDs encompasses crystallographic alignment, band alignment, specific orbital hybridization, and chemical bonding. As depicted in **Figure** **6**a, the interfaces can be categorized into two types: van der Waals (vdW) and non‐van der Waals interfaces.^[^ ^105^ ^]^ Van der Waals interfaces are mediated by vdW forces without the formation of chemical bonds, characterized by weaker interfacial binding and a more loosely structured interface.^[^ ^106^, ^107^ ^]^ Conversely, non‐van der Waals interfaces are formed through covalent bonds or other forces, involving stronger atomic binding and generally more compact interface structures.^[^ ^33^, ^106^ ^]^

#### 3.1.1 Van der Waals Interfaces

Van der Waals (vdW) interface structures between metals and 2D TMDs arise from short‐range interactions combined with induced dipole moments and polarizabilities (electrostatic forces).^[^ ^107^, ^108^ ^]^ Despite being the weakest interaction (typically ranging from 0.1 to 10 kJ mol⁻^1^, which is 2–3 orders of magnitude lower than ionic or covalent bonds), vdW forces (>10⁻^12^ N atom⁻^1^ or >10 N cm⁻^2^) play a crucial role in tightly contacted interfaces.^[^ ^30^ ^]^ They are significantly stronger than typical structural gravitational forces and are capable of holding large materials together (≈1–10 N cm⁻^2^ for materials 1 m thick), counteracting gravitational motion at the micro‐scale surface contacts between metals and semiconductors. Moreover, unlike covalent or ionic bonds, the strength of vdW forces does not rely on one‐to‐one chemical bonding and does not involve similar lattice symmetry or lattice constants between phases. Instead, it is predominantly influenced by intermolecular distances and the distribution of electronic clouds.^[^ ^27^, ^29^, ^109^ ^]^ This allows vdW interface strategies to be applicable across different material dimensions, including materials with varying crystal structures (crystallinity, lattice symmetry, lattice constants), electronic properties (metals, semiconductors, insulators, and superconductors), and dimensional forms (zero‐dimensional (0D), one‐dimensional (1D), two‐dimensional (2D), and three‐dimensional (3D)).^[^ ^110^ ^]^ The unique lattice structure and atomic crystal interface of 2D TMDs, coupled with their minimal chemical disorder, have garnered extensive interest in their vdW interactions with metals.^[^ ^111^, ^112^ ^]^

#### 3.1.2 Non‐Van der Waals Interfaces

The non‐van der Waals interfaces formed between metals and semiconductor 2D TMDs represent a complex and precise system, involving multiple levels of interactions. Initially, due to differences in lattice constants and orientations between metals and 2D TMDs, lattice distortions or defects may occur at the interface (Figure 6c,d), affecting electron transport and scattering between the phases. These distortions exhibit fluctuations beyond those of vdW forces,^[^ ^113^ ^]^ and display characteristics of covalent bonding.^[^ ^114^, ^115^ ^]^ Furthermore, the abundance of freely moving electrons in metals, whose wave functions extend broadly in space, can overlap with the valence electrons in 2D TMDs upon contact. This overlap facilitates the formation of covalent bonds between the metal and TMDs by sharing electron pairs across their orbitals (Figure 6e).^[^ ^33^, ^116^, ^117^ ^]^ From a lattice matching perspective, the formation of covalent bonds between metals and 2D TMDs is typically influenced by the degree of crystal lattice alignment, allowing metal and TMDs atoms to align more readily and enabling outer electron orbitals to overlap more easily, thus fostering covalent bond formation. Such covalent interactions contribute to the stability of the metal‐semiconductor interface, enhance electron transfer and injection, and thereby improve the reactivity and stability of electrocatalytic materials.^[^ ^47^, ^118^ ^]^

### 3.2 Interface Effects

**Figure 7:**
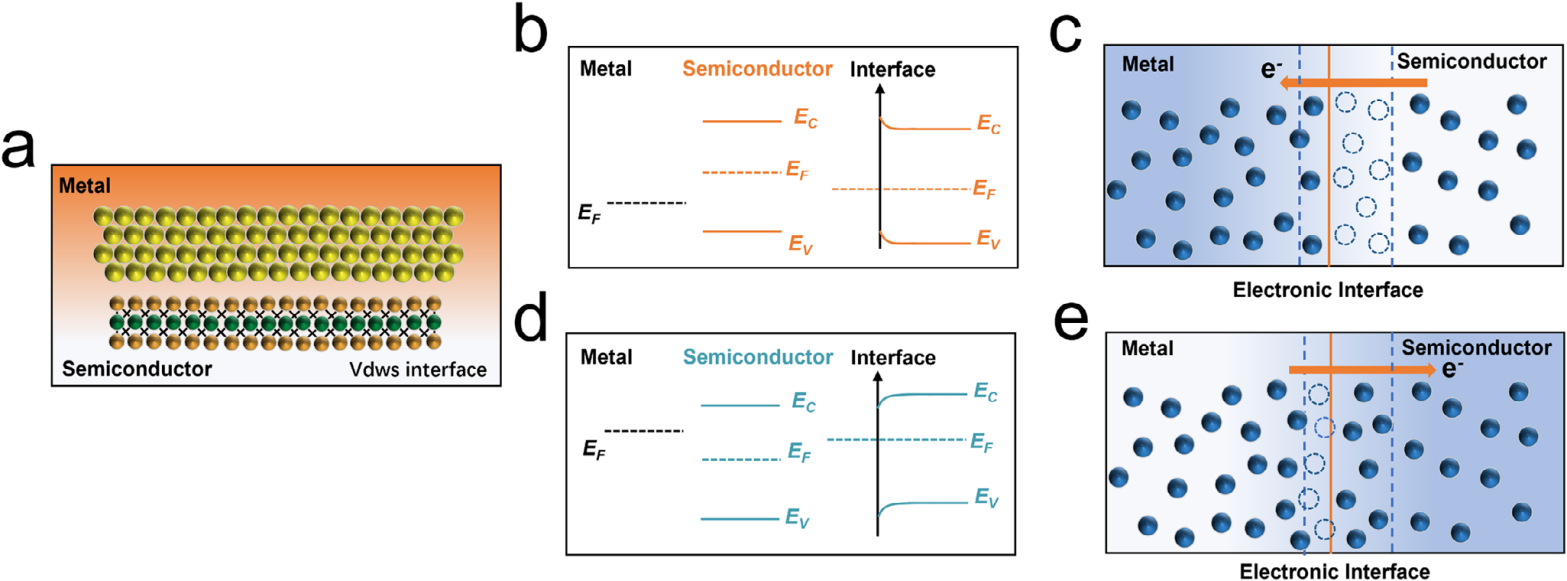
a) Schematic diagram of the van der Waals contact interface between metal and 2D TMDs; b,c) band structure diagram; d,e) electron transfer model, where (d) the interface is an electron depletion region and e) the interface is an electron enrichment region.

The interface effects between metals and 2D TMDs are influenced by the difference in energy between the metal work function and the semiconductor electron affinity (schottky barrier), as well as the interface lattice matching (fermi level pinning).^[^ ^33^, ^111^ ^]^ As depicted in **Figure** **7**a, for van der Waals contact, the interface effects between metals and 2D TMDs are predominantly governed by the inherent differences in their band structures. Even at zero bias, electrons will autonomously flow from the side with the relatively lower work function to the other, until the work functions on both sides of the interface equilibrate. This results in the formation of an electron depletion zone and an electron accumulation zone near the semiconductor side. When the metal work function exceeds the electron affinity of the 2D TMDs, electrons flow from the TMDs side to the metal, reducing the electron density on the TMDs side, causing upward band bending, and moving the fermi level downward, creating an electron depletion zone a “high‐resistance area” (Figure 7b,c). Conversely, if the electron affinity of the 2D TMDs is greater than the metal work function, electrons flow from the metal side to the TMDs, increasing the electron density on the TMDs side, resulting in downward band bending and moving the fermi level upward, thus creating an electron‐rich zone a “high‐conductivity area” (Figure 7d,e).

**Figure 8:**
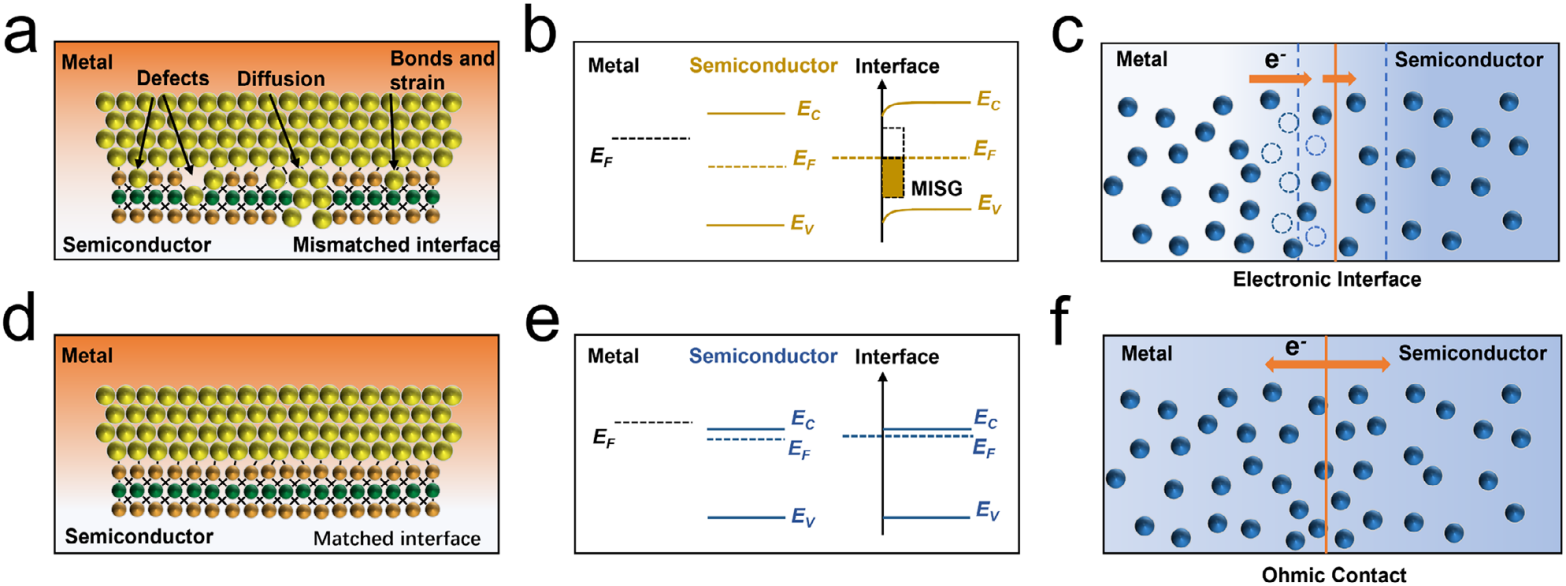
Schematic diagram of the non‐van der Waals contact interface between metals and 2D TMDs; a, d) defect‐induced interstitial state interface and high lattice symmetry interface; b, e) band structure diagram; c, f) electron transfer model.

As depicted in **Figure** **8**a, non‐van der Waals contacts, are significantly affected by various factors at the interface. The termination of the TMDs crystal structure results in incomplete covalent bonds and surface dangling bonds, while surface states formed through surface reconstruction, chemical bonding between metals and TMDs, and interdiffusion leading to lattice strain, as well as additional chemical disorder and defects inducing metal gap states, all contribute to severe fermi level pinning. This pinning drastically alters the interface characteristics, making it difficult to control the schottky barrier at the metal‐2D TMDs interface (Figure 8b,c), and increasing the contact resistance, which impedes the electron transfer process.^[^ ^119^ ^]^ In comparison, a high degree of lattice matching between the metal and TMDs maximizes the overlap of outer electron orbitals, reducing or eliminating the schottky barrier and forming ohmic contacts that allow free electron flow at the interface, without generating charge regions on either side (Figure 8e,f). This significantly improves the electron transfer rate and accelerates the electrocatalytic reaction process. In summary, by tuning the metal work function or designing high interface lattice matching, a controllable electron density region can be formed at the metal‐TMDs contact interface, or an electron transfer pathway can be constructed to reduce or eliminate interface resistance, thereby effectively promoting the adsorption/desorption behaviors of specific reactants in certain catalytic reactions.

## 4 Design Strategies for Ohmic Contact in Metal‐TMDs Systems

From a theoretical perspective, a crucial parameter for enhancing catalytic activity is the reduction of interface contact resistance, which optimizes the electron transfer process and accelerates reaction rates within specific electrocatalytic systems. The magnitude of contact resistance is primarily determined by the interfacial structure and the energy difference resulting from the schottky barrier, which arises from the disparity between the metal work function and the electron affinity of semiconductor 2D TMDs.^[^ ^33^, ^120^, ^121^ ^]^ This section reviews recent methods for controlling contact resistance, with a focus on interface contact structures and schottky barriers.

### 4.1 Metals Work Function and TMDs Phase Engineering

**Figure 9:**
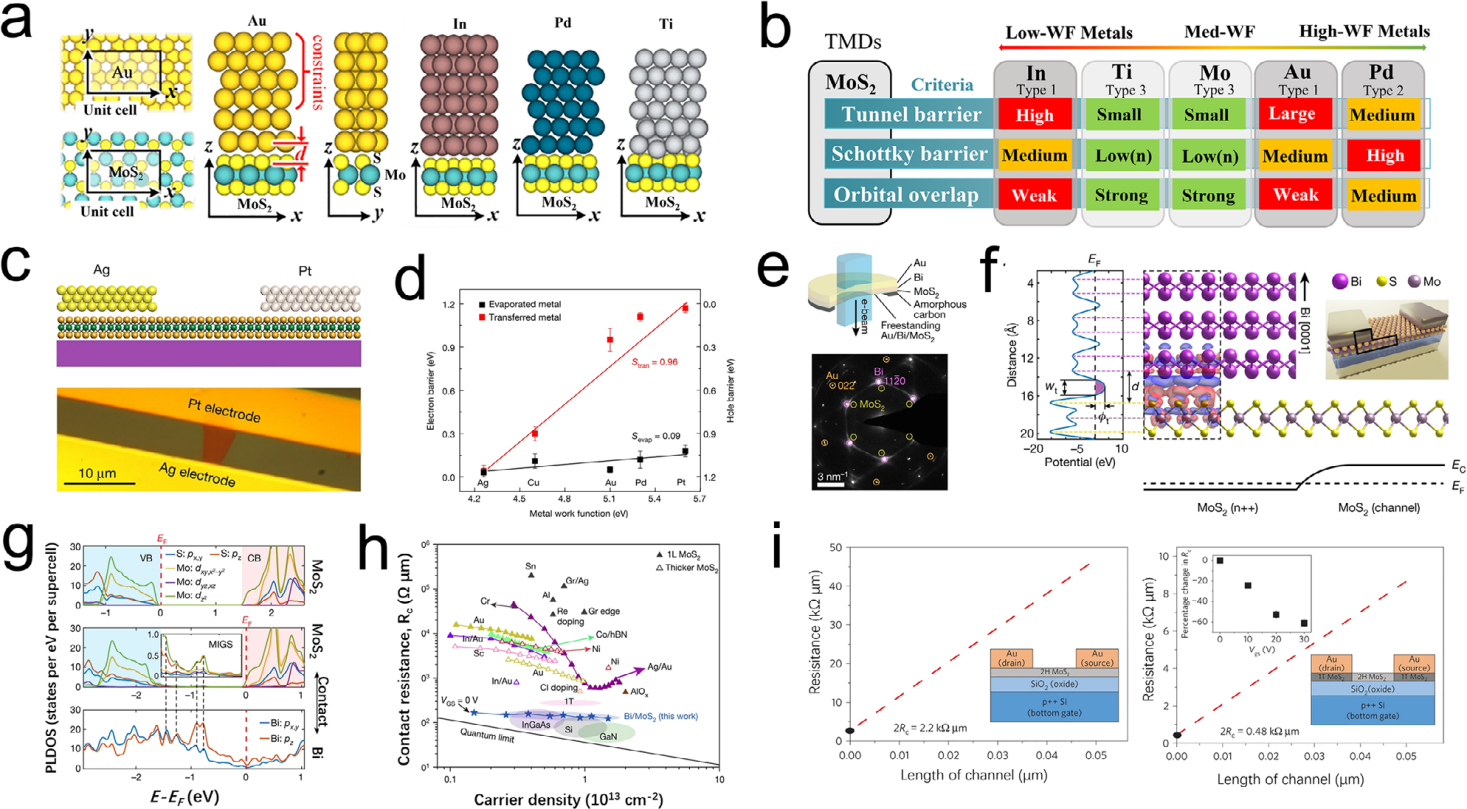
a) Optimized geometries of top contacts to MoS_2_: Au‐MoS_2_, In‐MoS_2_, Pd‐MoS_2_, Ti‐MoS_2_, Mo‐MoS_2_. d is defined as the physical separation (the z component of the nearest core‐to‐core distance between the metal atoms and the chalcogenide atoms). b) Summary of metal‐mTMDs top‐contact electron in jection efficiency, in terms of orbital overlap, schottky barrier, and tunnel barrier.^[^ ^47^ ^]^ Copyright 2014, American Physical Society. c) Schematic illustration and optical image of transferred asymmetric Ag and Pt electrodes on MoS_2_. d) Schottky barrier height for different transferred metals and evaporated metals.^[^ ^111^ ^]^ Copyright 2018, Springer Nature. e) Schematic of freestanding Au/Bi/MoS_2_ on a meshed amorphous carbon (a‐carbon) TEM grid for SAED patterns of MoS_2_ (3.6 cm^−1^), Bi (4.3 cm^−1^) and Au (6.8 cm^−1^) circled in yellow, pink and orange, respectively. f) The side view of Bi–MoS_2_ (upper right, with the area marked in the inset 3D render), and the corresponding electrostatic potential profile along the vertical direction (left). g) PLDOS of MoS_2_ before (upper panel) and after (middle panel) in contact with Bi (lower panel). The valence band is shaded in light blue and conduction band in light red. The fermi level (E_F_) is shifted from inside the gap (before Bi contact) to above the conduction band minimum (after Bi contact). Inset to middle, the magnified PLDOS in the bandgap, showing MIGS. h) State‐of‐the‐art contact technology for MoS_2_ transistors plotted as a function of n2D, showing the respective R_c_ of various semiconductor technologies (Si, III–Vs, and MoS_2_)^.[^ ^119^ ^]^ Copyright 2021, Springer Nature. i) The contact resistance (R_c_) at 2H is 1.1 k µm and at 1T is 0.2 k µm at zero gate bias. The inset shows the percentage drop in contact resistance with gate bias.^[^ ^124^ ^]^ Copyright 2014, American Institute of Physics.

From a structural perspective, 2D TMDs often terminate in atomically smooth, inert basal planes, making it challenging to form covalent bonds with metals. Consequently, contact between metals and TMDs is predominantly characterized by van der Waals forces in top‐contact configurations (**Figure** **9**a). In scenarios where interface disorder is not considered, charge injection into 2D TMDs heavily relies on the difference between the work functions of the metal and the 2D TMDs (Figure 9b). Thus, the simplest contact engineering strategy is to modify the work function of the contact metal or manipulate the crystal phase of TMDs to reduce the height of the schottky barrier, thereby forming a low‐resistance contact interface.^[^ ^33^, ^122^ ^]^ For example, as depicted in Figure 9c,d, Liu et al. employed a straightforward physical strategy of laminating flat metal films with varying work functions onto 2D MoS_2_. By exploring the correlation between the metal work functions and schottky barrier heights, they verified the fundamental limits of the ideal metal‐semiconductor junction. Results indicated that, compared to chemically bonded metal‐MoS_2_ interfaces, transferred metal‐MoS_2_ exhibited atomically clean interfaces, free from interfacial gap states caused by chemical disorder and defects during deposition. Experiments with low work function Ag (W_Ag_ = 4.3 eV), medium work function Cu (W_Cu_ = 4.6 eV), and high work function Au (W_Au_ = 5.3 eV) showed that vdW contacts in MoS_2_ transistors exhibit tunable schottky barrier heights determined by the metal work functions, with contacts with Ag demonstrating the lowest contact resistance and schottky barrier height.^[^ ^111^ ^]^ Wang et al. achieved ultraclean van der Waals contacts between 10 nm thick low work function metal (covered by 100 nm thick Au electrodes) and single‐layer TMDs (MoS_2_, WS_2_, NbS_2_) through chemical vapor deposition and evaporation strategies, exploring the interfacial contact effects between 3D metals and single‐layer 2D TMDs. The results showed that the contacts between WS_2_, WSe_2_, and MoS_2_ with indium alloys exhibited no detectable chemical interactions, while extremely low contact resistance and near‐ideal band alignment further confirmed the defect‐free nature of WS_2_, WSe_2_, and MoS_2_ contacts with indium alloys.^[^ ^122^ ^]^ Shen et al. proposed using contacts between semi‐metals and semiconductors to suppress metal‐induced gap states (MIGS) and reduce contact resistance. Contact tests between chemically vapor‐deposited single‐layer MoS_2_ and Bi, Ni, and Ti showed that MIGS were effectively suppressed in TMDs with low work function semi‐metal Bi, spontaneously forming orbital degeneracy that promoted the formation of ohmic contacts (contact resistance: 123 Ω µm) (Figure 9e–h).^[^ ^119^ ^]^ To investigate the role of different structural phases of 2D TMDs during their interaction with metals, R. Kappera et al. immersed monolayer 2H‐MoS_2_ in n‐butyllithium solution, resulting in a 60–70% transformation of the treated area to the 1T phase, thereby forming a 1T/2H mixed‐phase MoS_2_. This phase transition reduced the work function of MoS_2_, eliminating the schottky barrier formed at the Au‐MoS_2_ interface and consequently enabling the formation of contacts with low contact resistance (200 Ω µm⁻^1^) (Figure 9i).^[^ ^123^, ^124^ ^]^ Suyeon Cho and colleagues demonstrated a similar process using low‐power laser‐induced local phase transformation from semiconducting 2H‐phase MoTe_2_ to twisted 1T’ phase, which reduced the schottky barrier height from 200 meV to 10 meV with Au contacts, reducing interface resistance and thus enhancing electron mobility.^[^ ^47^ ^]^ Although controlling the work function of contact metals or semiconductors can reduce contact resistance and promote directional electron transfer, contacts primarily based on van der Waals interfaces struggle to maintain consistent performance under high current densities over extended periods, making them unsuitable for large‐scale production.

### 4.2 Edge Contact Engineering

**Figure 10:**
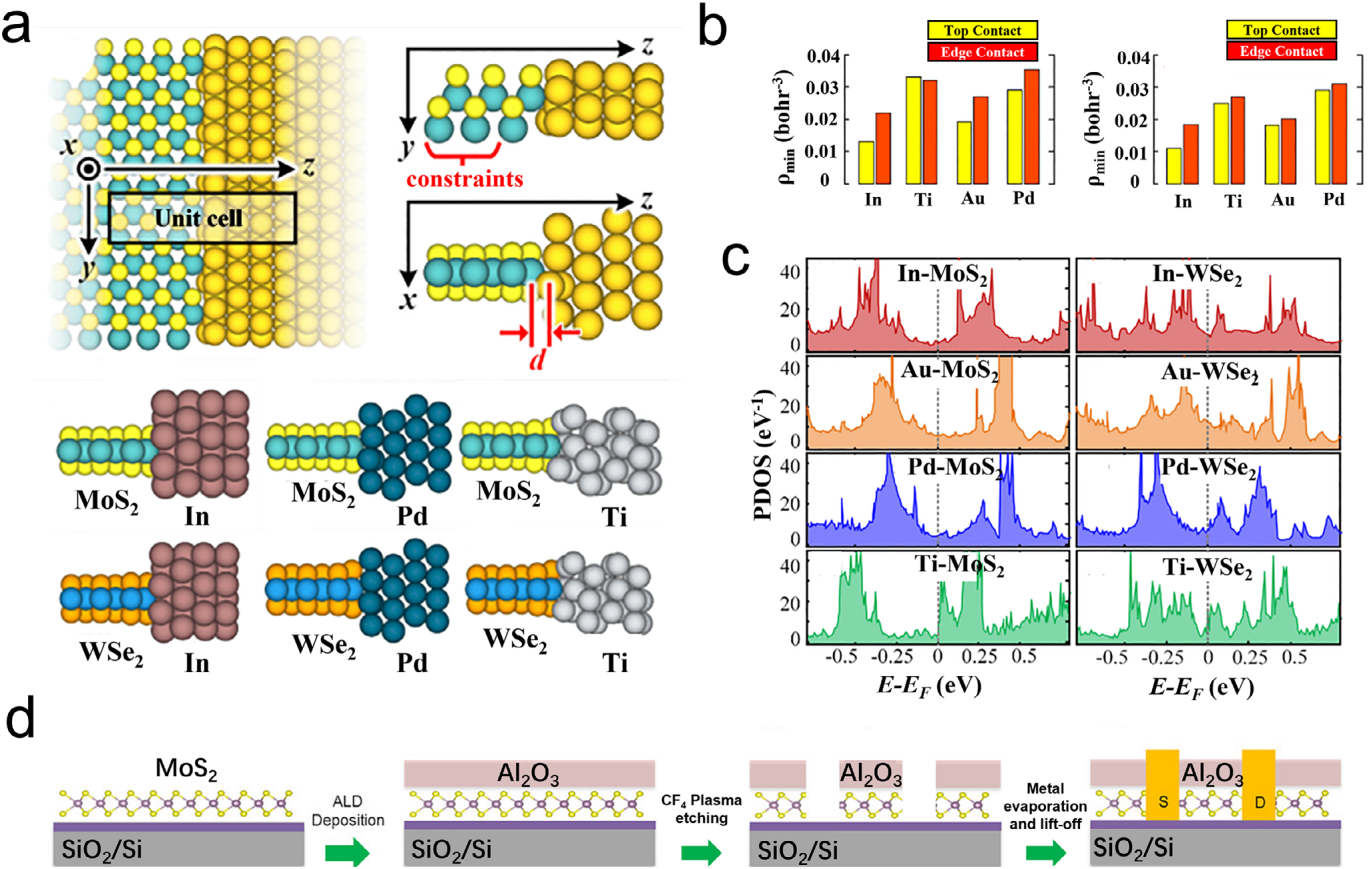
a) Optimized geometries of edge contacts: Au‐MoS_2_ (in different views), In‐MoS_2_, Pd‐MoS_2_, Ti‐MoS_2_, Au‐WSe_2_ (in different views), In‐WSe_2_, Pd‐WSe_2_, and Ti‐WSe_2_. b) Minimum of average electron density values in the x‐y plane in Figure 9a at the interfaces for all top and edge contacts. c) PDOS of mTMDs near *E_F_* of edge contacts to MoS_2_ and WSe_2_. Because of orbital overlaps (covalent bonds) at the interfaces, all the mTMDs in edge contacts have overlap states in the original band gaps and near *E_F_*, so that mTMDs are metallized by edge contacts.^[^ ^47^ ^]^ Copyright 2014, American Physical Society. d) Schematic illustration of the fabrication procedure for creating edge contacts on MoS_2_ with Al_2_O_3_ cover‐layer.^[^ ^127^ ^]^ Copyright 2016, Wiley‐VCH.

Numerous studies have demonstrated that the mode of interface contact between metals and TMDs significantly influences their electronic properties and carrier transport mechanisms. Traditional top contacts on TMDs, characterized by wide tunnel barriers and low covalency at the interface, provide a disadvantageous configuration for out‐of‐plane carrier transport, impeding effective electron transmission across the interface.^[^ ^34^ ^]^ In contrast, edge contacts offer a superior alternative, characterized by higher covalency and narrower tunnel barriers. This configuration facilitates greater orbital overlap and simplifies in‐plane carrier migration within the TMDs layers, enhancing overall electronic efficiency and functionality.^[^ ^116^, ^125^ ^]^ For instance, significant improvements in interface electron transfer behaviors can be achieved by optimizing the metal‐TMDs contact area. Y. Matsuda et al. (2007) employed DFT calculations to explore the contact resistance at metal‐graphene edges, revealing that the resistance is influenced by cohesive forces and electron interactions at the interface. The coupling between metal d‐orbitals and carbon pπ‐orbitals is crucial in determining the contact resistance, with edge contacts on graphene involving contributions from C ps orbitals, thereby increasing cohesive coupling and improving electron transport properties compared to traditional top contacts.^[^ ^117^ ^]^ For TMDs, the advantages of edge over top contacts are clear: edge contacts exhibit shorter bonding distances, higher orbital overlap, minimal tunnel barrier widths, and larger contact areas, all of which promote electron interactions between the metallic substrate and the semiconductor TMDs, benefiting in‐plane carrier injection.^[^ ^116^ ^]^ As depicted in **Figure** **10**a, Kang et al. systematically studied the contacts between single‐layer TMDs edges and various metals such as In, Ti, Au, Pd, Mo, and W, showing that physical separation (𝑑) at edge contacts was consistently less than at top contacts, with atomic overlap between the metal and the sulfide observed at every edge contact. These edge contacts demonstrated strong orbital overlap effects, the absence of schottky barriers, and lower tunnel barriers, leading to higher electron injection capabilities and reduced contact resistance (Figure 10b,c).^[^ ^47^ ^]^

For multilayer 2D materials, these effects are magnified, as each layer can form edge contacts, effectively enhancing charge injection deep into the 2D material.^[^ ^126^ ^]^ Edge contacts also benefit scalability, as overlapping regions between metal and TMDs are no longer necessary. However, fabricating edge contacts, especially for thicker 2D layers, remains challenging with traditional deposition techniques.^[^ ^34^ ^]^ One approach involves etching away an insulating layer beneath the contact area prior to metal deposition, preventing direct top contact of deposited TMDs with the metal, thus favoring edge contacts. As demonstrated by Y. Chai et al., using ALD deposition techniques and CF_4_ plasma to etch grooves in a passivation layer of Al_2_O_3_, multilayer MoS_2_ was exposed at the edges to directly contact Al_2_O_3_/SiO_2_, as shown in Figure 10d.^[^ ^127^ ^]^ Marcos H. D. Guimarães and colleagues conducted similar work using metal‐organic chemical vapor deposition on etched single‐layer graphene to prepare edge contacts for TMDs (WS_2_ and MoS_2_). Their findings indicated that graphene and TMDs layers were laterally connected with wafer‐level uniformity, devoid of van der Waals gaps, tunnel barriers, or multiple overlapping top contacts, achieving an average contact resistance as low as 30 kΩ·µm. Edge contact technology between metals and 2D TMDs significantly enhances electron transfer efficiency and interface stability. However, the complex fabrication processes and challenges in achieving uniform and consistent edge contacts at large scales, along with the potential defects at the edges, may hinder its large‐scale application and long‐term stability.^[^ ^128^ ^]^

### 4.3 Interface Doping Engineering

**Figure 11:**
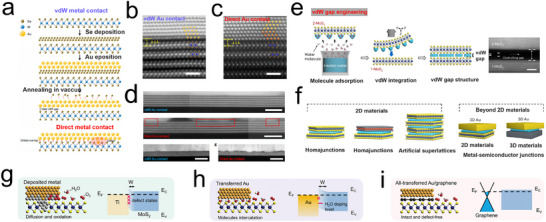
WSe_2_ with vdW and direct Au contact. a) Schematic of the sample cross‐sections during the fabrication of vdW and direct Au contact on WSe_2_; b) HAADF‐STEM image of the cross‐section of WSe_2_ with vdW Au contact; c) STEM images of WSe_2_ cross‐section with direct Au contact; d) Enlarged view of the interface area between Au and WeS_2_ van der Waals contact and direct contact.^[^ ^132^ ^]^ Copyright 2022, Springer Nature. e) Schematic illustrations of the vdW gap engineering achieved by adsorption of water molecules. f) Expandable systems for vdW gap engineering, including 2D/2D homojunctions, 2D/2D heterojunctions, 2D artificial superlattices, and 2D/3D and 3D/3D heterojunctions. Atomic structures, band structures, and comprehensive performance for MoS_2_ transistors contacted with different kinds of electrodes.^[^ ^134^ ^]^ Copyright 2024, Springer Nature. g) Deposited Ti/Au electrode. h) Transferred Au electrode. i) All‐transferred Au/graphene electrode.^[^ ^133^ ^]^ Copyright 2023, Wiley‐VCH.

The vdW gap, characteristic of the interface in van der Waals contacts between metals and 2D TMDs, plays a critical role in determining the contact resistance and electron transport efficiency between the metal and the 2D TMDs.^[^ ^129^ ^]^ Current strategies to modulate the vdW gap typically involve intercalation and doping techniques, wherein angstrom‐level vdW gaps allow for the confinement of molecules or ions on an ultra‐small scale, thereby enabling the tailoring of material properties. Furthermore, due to the anisotropic bonding in their structure (covalent bonds within the layers and vdW interactions between them), 2D TMDs exhibit weak interlayer interactions and a high sensitivity to environmental changes, making them highly suitable for guest (molecule, atom, ion) intercalation.^[^ ^27^, ^130^ ^]^ By preserving the host structure of the TMDs without disrupting the intralayer covalent bonds and by restricting the doping substances, guest materials can be inserted into the layered TMDs.^[^ ^32^ ^]^ This insertion further alters the host material's interfacial band structure and electronic interactions, thereby reducing contact resistance.^[^ ^131^ ^]^ For instance, as depicted in **Figure** **11**a–d, Gihyeon Kwon and colleagues employed Se as a buffer layer in their metal deposition process to create a series of interaction‐free and defect‐free van der Waals contacts between metals and 2D materials. Using Au‐WSe_2_ as an example, they demonstrated the impact of interface structure on electron mobility between van der Waals contact and direct metal contact. Their results showed that direct contact between WSe_2_ and Au led to the formation of multiple new electronic levels within the bandgap of WSe_2_, exhibiting a significant electron exchange density. In contrast, the interface gap in the van der Waals contact between Au and WSe_2_ prevented orbital overlap, resulting in less electron exchange.^[^ ^132^ ^]^ Subsequently, Jiang et al. conducted similar research by inserting an atomic layer of yttrium as a buffer layer between the metal and MoS_2_. This buffer layer prevented lattice degradation at the van der Waals interface and the occurrence of Fermi level pinning, reducing contact resistance between the metal and MoS_2_ and enhancing the transfer of charge carriers from the metal electrode to the semiconductor MoS_2_.^[^ ^46^ ^]^

Building on a deeper understanding of interface modifications, Wu and colleagues further explored the impact of direct graphene intercalation on the properties of metal‐semiconductor interfaces through layered material approaches. They first transferred high‐quality single‐layer graphene to a transitional substrate, then transferred Au pads onto the graphene surface, and finally moved the Au/graphene pads onto single‐layer MoS_2_. The results indicated that the presence of a defect‐free graphene buffer layer preserved the electrode‐channel interfaces intact and robust, reduced the schottky barrier height of the t‐Au/graphene‐MoS_2_ electrodes, leading to lower contact resistance and higher carrier mobility (contact resistance of 4.7 kΩ µm at a carrier density of 3.2 × 10^12^ cm⁻^2^). Furthermore, the presence of a defect‐free hydrophobic graphene buffer layer prevented metal diffusion from the electrode to MoS_2_ and the insertion of water molecules at the t‐Au/graphene‐MoS_2_ interface, thereby ensuring the structural stability of the electrodes (Figure 11g–i).^[^ ^133^ ^]^

Addressing the precise control of the van der Waals gap height, Liu et al. proposed using H₂O molecule intercalation to adjust the interlayer van der Waals gap of MoS_2_, with water molecule intercalation enabling control from anhydrous gaps to gaps as high as 30.6 Å (Figure 11e). This strategy provides a universal approach for controlling the height of the van der Waals gap in 3D metal/2D TMDs interfaces, allowing for the free adjustment of interlayer coupling, conductivity, chirality, and topological structures between 2D van der Waals materials and other materials, thereby facilitating their development in the fields of solid‐state electronics and energy catalysis (Figure 11f).^[^ ^134^ ^]^ However, while interface doping strategies can optimize electron transfer pathways and interfacial interactions, excessive strain induced by defects or uneven doping may negatively affect the catalytic performance and long‐term stability of electrocatalytic reactions. Therefore, this strategy is better suited for systems that require specific modulation, such as exfoliation‐based preparation of 2D materials or the precise control of interfaces and assisted deposition processes in advanced materials.

### 4.4 Orbital Hybridization and Interface Bonding Engineering

**Figure 12:**
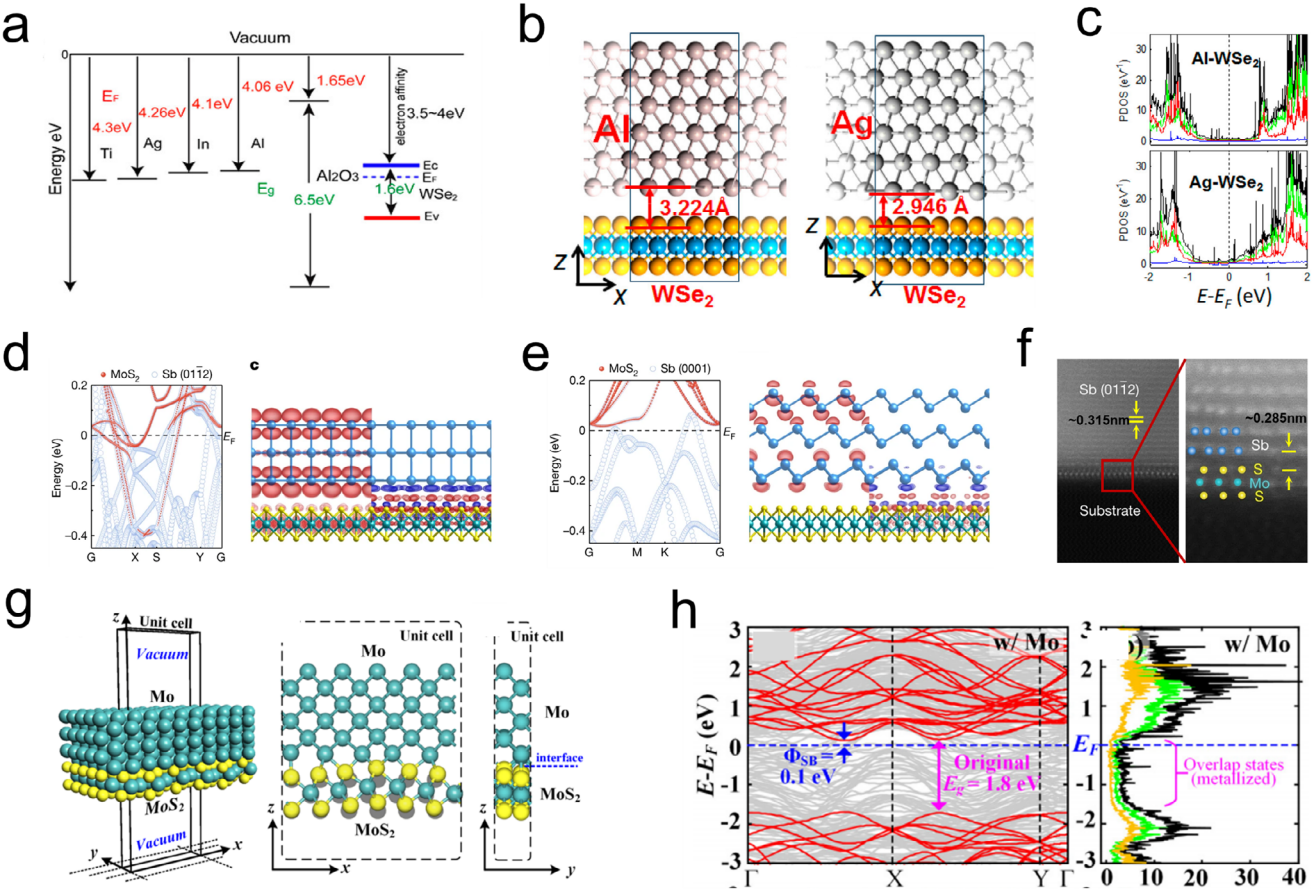
a) Band alignments of some potential contact metals and Al_2_O_3_ with respect to that of WSe_2_. E_C_ and E_V_ represent the conduction and valence band edges of WSe_2_, respectively. b) Side view of the relaxed contact region at the interface between WSe_2_ and Al (111) and Ag (111) surfaces. c) PDOS of W and Se electron orbitals for Al‐WSe_2_ and Ag‐WSe_2_ systems (from top to bottom). The green, blue, red, and black curves represent the d orbitals of tungsten (W) atoms, the sp orbitals of W atoms, the sp orbitals of selenium (Se) atoms, and the total PDOS of WSe_2_, respectively.^[^ ^136^ ^]^ Copyright 2013, American Chemical Society. d) Atomic projected electronic band structure of Sb (0001)‐MoS_2_ contact and charge density near junction *E_F_*. e) Atomic projected electronic band structure of Sb (0112)‐MoS_2_ contact and charge density near junction E_F_ (red, positive; blue, negative).^[^ ^126^ ^]^ Copyright 2023, Springer Nature. f) (Left) Cross‐section HAADF‐STEM image of the Sb (0112)–MoS_2_ contact. Scale bar, 2 nm. (Right) Zoom‐in atomic‐resolution image from the red box in c. The Mo, S and Sb atoms are overlayed on the image. The vdW gap of 0.285 nm is marked. Scale bar, 1 nm. g) Unit cell of Mo‐MoS_2_ system (top‐contact) in 3D view, side view (x‐z), and side view (y‐z). h) Band structure and orbital PDOS of pristine MoS_2_ and Mo‐MoS_2_ system (relative to the unit cell of (g)).^[^ ^118^ ^]^ Copyright 2014, American Institute of Physics.

As previously mentioned, the majority of practical contact structures involve the edges and top surfaces of 2D TMDs. However, the inherent surface properties of 2D TMDs and the challenges associated with forming edge contacts have limited the large‐scale development of these materials.^[^ ^33^ ^]^ Further research into interface interactions has revealed that orbital hybridization and chemical bonding between metal atoms and 2D TMDs can significantly reduce contact resistance, potentially enabling the formation of true ohmic contacts.^[^ ^119^ ^]^ Near the interface between metals and 2D TMDs, adjacent atomic orbitals can hybridize to form new mixed orbitals.^[^ ^47^ ^]^ This orbital hybridization leads to the emergence of new energy levels at the interface, prompting adjustments in the electronic structure and increasing the overlap of electron density between atoms, which facilitates electron transfer and enhances polarization effects.^[^ ^45^ ^]^ Moreover, the extent of orbital hybridization impacts the stability of covalent bonds; higher degrees of hybridization facilitate the formation of covalent bonds, thereby enhancing the mechanical stability of the material.^[^ ^103^, ^135^ ^]^ For instance, as depicted in **Figure** **12**a, Liu et al. investigated contacts between In, Ag, Al, Ti, and WSe_2_, finding that metals with d‐orbitals such as In, Ag, and Ti could hybridize with the d‐orbitals of Se and W in WSe_2_, thereby facilitating electron injection and forming ohmic contacts. Notably, Ag, with its low work function, exhibited the lowest contact resistance and highest electron mobility (205 µA µm⁻^1^ and 202 cm^2^(V·s) ⁻^1^) when contacted with WSe^2^. Conversely, Al, lacking d‐orbitals, demonstrated smaller orbital overlap and lower interfacial electron density with WSe_2_, resulting in higher interface resistance (Figure 12b,c).^[^ ^136^ ^]^ Li and colleagues, utilizing electron beam evaporation, prepared contacts between Sb with different crystal orientations and single‐layer MoS_2_ (Sb (0001)‐MoS_2_ and Sb (0112)‐MoS_2_), thereby corroborating this theory. In Sb (0112)‐MoS_2_, multiple hybridization bands composed of Mo d‐orbitals and Sb p and s orbitals intersected the fermi level (*E_F_*). There was a significant real‐space overlap of Mo d‐orbitals with Sb pz orbitals in the vertical direction, leading to strong hybridization and driving the interface resistance toward the quantum limit, thereby achieving ohmic contact with a contact resistance of 42 Ω µm. Conversely, no significant electronic localization was observed between Mo d‐orbitals and Sb (0001) p orbitals (Figure 12d–f).^[^ ^126^ ^]^

To further explore the impact of covalent bonding on ohmic contacts, Kang et al. employed electron beam lithography to deposit mechanically exfoliated MoS_2_ onto a Mo substrate (Mo‐MoS_2_). This led to the formation of Mo‐S covalent interface bonds between the S atoms in MoS_2_ and the contacting Mo atoms, causing periodic deformation and fracturing of the MoS_2_ structure (Figure 12g). The substantial band overlap effect resulted in the disappearance (metalization) of the MoS_2_ bandgap upon contact with Mo, with a reduction in the fermi level (Figure 12h). Consequently, the high‐quality contact interface formed between Mo and single‐layer MoS_2_ displayed zero tunnel barrier and zero schottky barrier. Due to strong fermi level pinning, the underlying MoS_2_ also exhibited an extremely low schottky barrier height (0.1 eV), enhancing electron injection efficiency and reducing contact resistance.^[^ ^118^ ^]^ Similarly, Luo et al. grew single‐layer TMDs on 3D nanoporous gold (NPG) via chemical vapor deposition (CVD). The involvement of sulfur led to the reconstruction of the Au {001} surface, forming an unconventional Au_4_S_4_ superstructure on the top layer of Au {100}. This reconstructed Au surface disrupted the anti‐symmetry of S‐Mo‐S in the top 1H MoS_2_, reducing the distance between Au and Mo, and enhancing the vdW interaction between the Au_4_S_4_ interface phase and the TMDs single layer. This transformed the n‐type TMDs‐Au Schottky contact into a p‐type, lowering the schottky barrier height from 0.89 eV to 0.45 eV and promoting the charge transfer process.^[^ ^137^ ^]^ Compared to other strategies, the formation of orbital hybridization or covalent bonds between metals and TMDs facilitates efficient electron transfer. Additionally, the strong interfacial interactions promote sustained catalytic activity over extended periods, making these composites highly promising candidates for industrial electrocatalysis applications. However, the complex processes involved in covalent bond formation may present challenges for large‐scale implementation. This approach is often integrated with theoretical calculations and in situ preparation techniques to gain deeper insights into the structural evolution during the process.

## 5 Interface Effects in Electrocatalytic Processes

As previously mentioned, inspired by the in‐depth analysis of interface behaviors between metals and 2D TMDs in the solid‐state electronics field, the development of ohmic contact‐type composite electrocatalysts in the electrochemical domain hinges on establishing efficient pathways for electron transfer between metals and 2D TMDs.^[^ ^138^ ^]^ By manipulating the interface contact resistance to control the charge transfer rate and reduce reaction barriers, catalytic activity and reaction selectivity can be optimized. Additionally, leveraging electronic interactions between interface contact atoms, orbital overlap effects, or specific orbital hybridization processes can further enhance interface stability, leading to more efficient and stable electrocatalytic processes.^[^ ^139^ ^]^ The interface effects in metal‐2D TMDs electrocatalytic materials, differentiated by the mode of contact and interface structure, are categorized into support and synergistic effects. This section will focus on these two key interfacial effects and their applications in electrochemical energy conversion, while summarizing the current applications of ohmic contact‐based catalysts in catalytic reactions.

### 5.1 Support Effects

**Figure 13:**
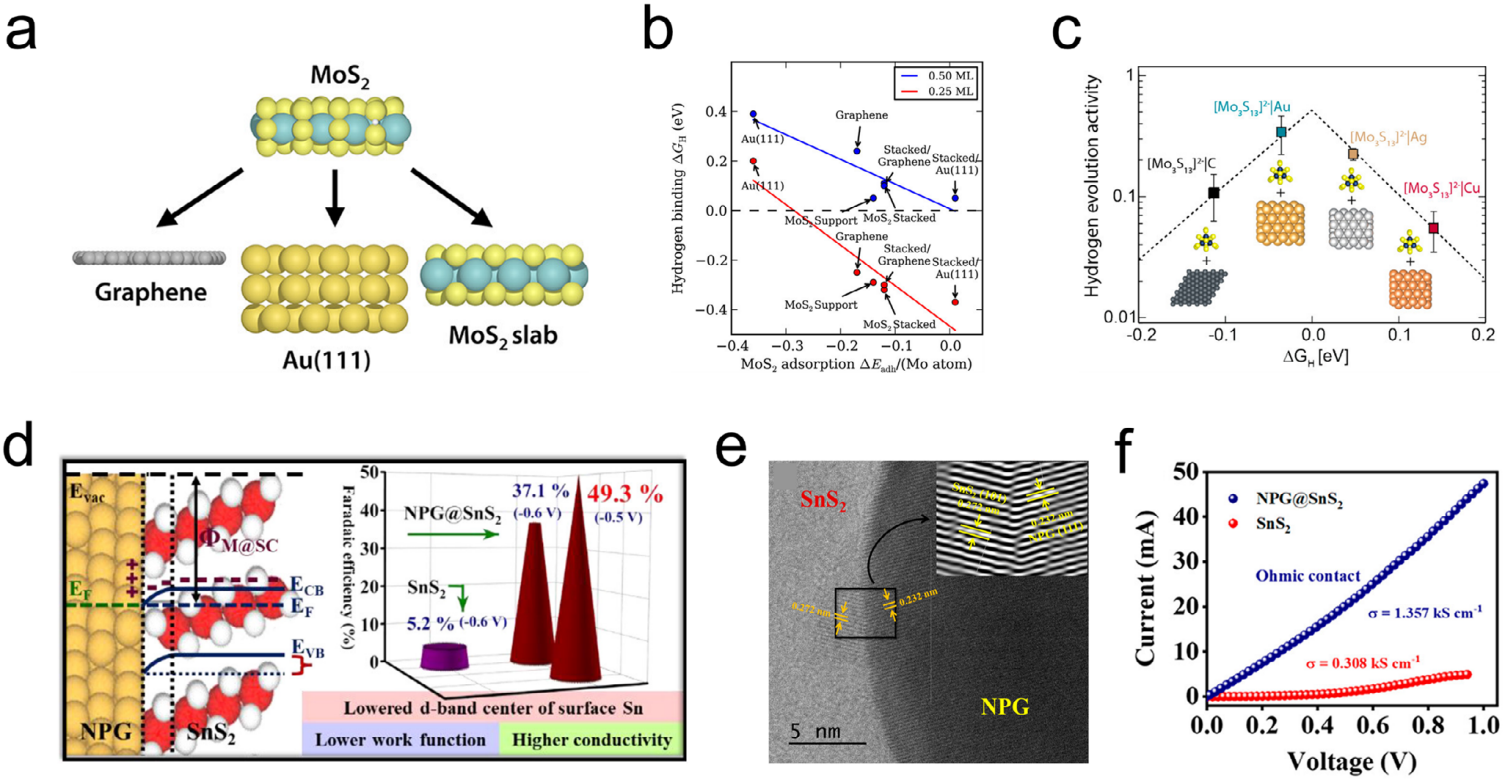
a) Schematic diagram of MoS_2_ loaded on different carriers. b) The change of Gibbs free energy of H‐absorption on the Mo‐edge at each coverage of MoS_2_ supported by different support.^[^ ^140^ ^]^ c) The TOF values at η = 200 mV as a function of ΔG_H_.^[^ ^141^ ^]^ Copyright 2017, American Chemical Society. d) Energy band structure of NPG@SnS_2_ and Faradaic efficiency of ammonia formation at −0.5 V. e) HRTEM; (inset of e) IFFT of NPG@SnS_2_ showing distinct layers of NPG and SnS_2_ at the heterojunction. f) I–V (conductivity) measurements of NPG@SnS_2_.^[^ ^142^ ^]^ Copyright 2021, American Chemical Society.

The choice of metal substrate can significantly influence the electronic properties and thermal stability of surface TMDs catalysts, thereby substantially impacting their catalytic activity.^[^ ^136^, ^137^ ^]^ Support effects offer a straightforward method to optimize the binding energies of reaction intermediates, enhancing electrocatalytic performance. In 2014, Charlie Tsai and colleagues utilized DFT to analyze how different substrates affect the HER activity at the MoS_2_ edge sites. Their studies compared systems including MoS_2_, MoS_2_‐Au, and MoS_2_‐graphene. As illustrated in **Figure** **13**a,b, the hydrogen adsorption free energies at the Mo edge with 0.25 monolayer (ML) H coverage were significantly increased by 0.56 eV and 0.18 eV for MoS_2_‐Au and MoS_2_‐graphene, respectively, compared to unsupported MoS_2_. This effect indicated that the support provided by Au (111) or graphene influenced only the top layer of MoS_2_, demonstrating a short‐range support effect.^[^ ^48^ ^]^ In the same year, Zhang and colleagues discovered that MoS_2_ exhibits strong interactions with gold foil substrates, facilitating effective electron transfer between the gold substrate and the catalytic active sites at the edges of MoS_2_. This excellent electron coupling between Au and MoS_2_ minimized charge transfer resistance, enabling the composite catalyst to achieve a high exchange current density of 38.1 µA cm⁻^2^ in the HER.^[^ ^140^ ^]^ Subsequently, in 2017, the support effect was employed as an effective strategy to tune the ΔG_H_ of [Mo_3_S_13_]^2^⁻ cluster catalysts, optimizing their inherent HER activity. The findings indicated that the carrier had a significant regulatory effect on the activity of [Mo_3_S_13_]^2^⁻ clusters, with [Mo_3_S_13_]^2^⁻‐Au displaying the lowest initial HER overpotential (−0.15 V) and the highest turnover frequency (TOF) of 0.47 s⁻^1^ at −200 mV relative to RHE (Figure 13c).^[^ ^141^ ^]^ Ashmita Biswas and colleagues, based on interface engineering strategies, deposited metallic Sn on the surface of cubic nanoporous gold (NPG) via electron beam deposition, followed by sulfurization at 450 °C, fabricating a heterostructured NPG@SnS_2_ electrocatalyst (Figure 13d,e). Experimental results revealed that the direct contact between the metal NPG and n‐type semiconductor SnS_2_ led to a redistribution of charges and bending of energy bands, elevating the overall Fermi level of the material, lowering the work function, and enhancing the conductivity of NPG@SnS_2_ (Figure 13f). Additionally, the repositioning of the d‐band center of surface Sn atoms weakened competitive H adsorption processes, promoting N_2_ adsorption at Sn sites. This enabled NPG@SnS_2_ to achieve an ammonia formation Faradaic efficiency of 49.3% at −0.5 V.^[^ ^142^ ^]^ These examples underscore the critical role played by metal substrates in altering the electronic state, dispersibility, stability, and reaction dynamics of surface catalysts, thereby serving a pivotal function in electrocatalytic reactions.

### 5.2 Synergistic Effects

**Figure 14:**
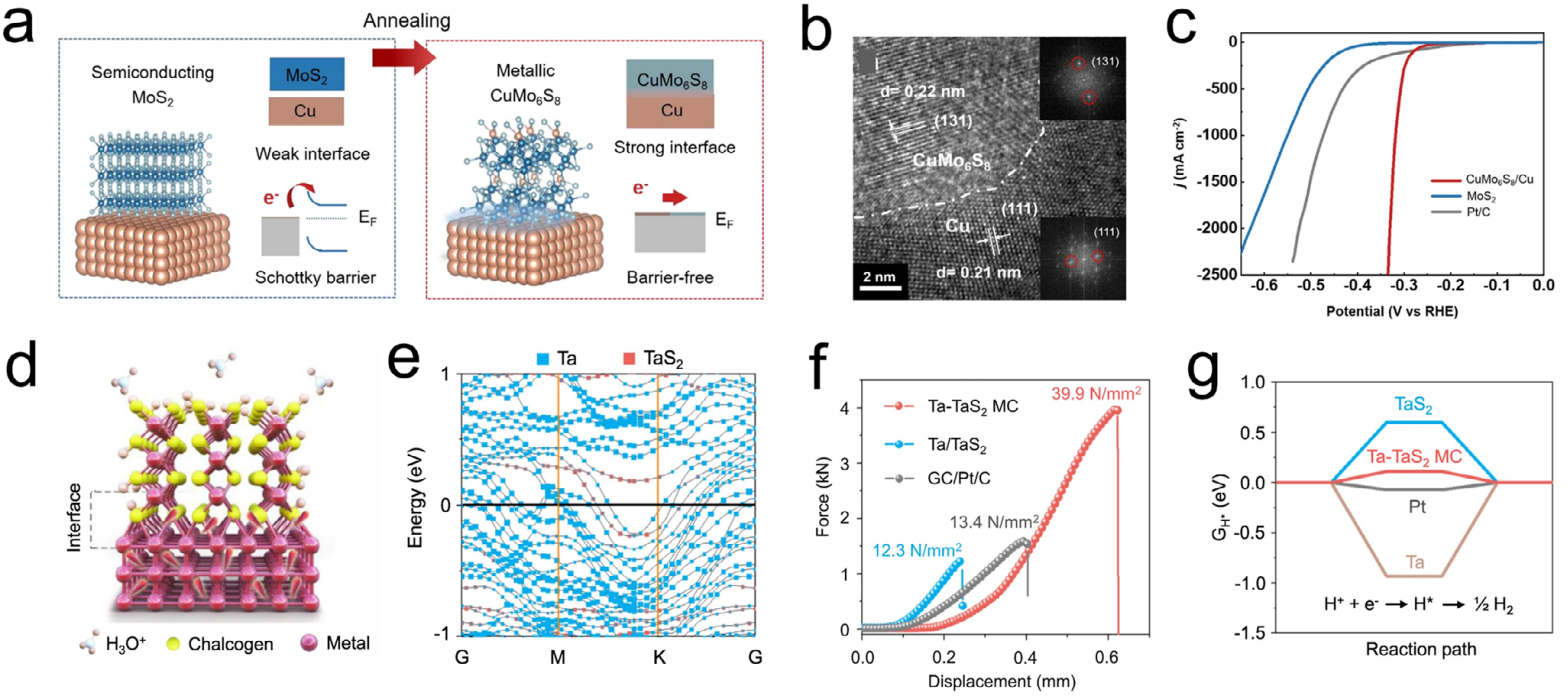
a) A schematic showing the preparation process of CuMo_6_S_8_/Cu electrode. b) TEM image of the interface between the CuMo_6_S_8_ layer and the Cu substrate. The inset is the corresponding fast Fourier transform patterns of CuMo_6_S_8_ (top) and Cu (bottom). c) Polarization curves of three electrodes including CuMo_6_S_8_/Cu, MoS_2_, and Pt/C.^[^ ^135^ ^]^ Copyright 2022, Springer Nature. d) Atomic structure of the Ta‐TaS_2_ MC. e) Ta and TaS_2_ band structures. f) Force‐displacement curves for Ta‐TaS_2_ MC, Ta/TaS_2_, and a commercial Pt/C bound to glassy carbon for comparison. g) Hydrogen absorption free energy diagram of Ta, TaS_2_, Ta‐TaS_2_ MC, and Pt catalysts.^[^ ^103^ ^]^ Copyright 2021, Springer Nature.

Unlike support effects, synergistic effects in multi‐component electrocatalytic systems arise from strong bond coupling, orbital hybridization, or electronic interactions between different materials, facilitating efficient pathways for electron transfer between metals and 2D TMDs.^[^ ^143^, ^144^, ^145^ ^]^ This interaction leads to the reconstruction of active centers, with the catalytic performance surpassing the simple sum of individual components. The interplay between the TMDs' active components and metal substrates, and its significant impact on electrocatalytic HER activity, has become a focal point of recent research.^[^ ^48^ ^]^ As illustrated in **Figure** **14**a, Hyunjung Shin and Liu et al. developed a highly mechanically stable new type of electrocatalyst by growing MoS_2_ nanosheets vertically embedded into a self‐supporting Cu foil. The resulting MoS_2_ nanosheets were embedded into a Cu_2_S matrix (Figure 14b), where the formation of chevrel Cu_x_Mo_6_S_8_ clusters between MoS_2_ and Cu_2_S led to induced asymmetric charge transfer within the layered framework. During surface reactions, the metal substrate, acting as a self‐supporting electrode, injects electrons into the CMS (Cu‐Mo‐S) layer, enhancing internal carrier transport within CMS. Furthermore, MoS_2_ nanosheets generated within CMS elevated the overall material's fermi level, further reducing barrier heights and enhancing the HER process. Electrochemical testing showed that the synthesized CMS exhibited a low overpotential of ≈334 mV at a current density of 2500 mA cm⁻^2^, with sustained stability exceeding 100 h (Figure 14c).^[^ ^95^, ^135^ ^]^ Liu et al. demonstrated that in situ grown TMDs on homo‐metallic substrates enable direct electron injection from the substrate metal into the active TMDs layer, effectively eliminating the high electronic barriers caused by van der Waals gaps. As shown in Figure 14e,f, the extensive and dispersive electronic states contributed by both Ta and TaS_2_ span across the system's fermi level, confirming the excellent electronic transfer capability at the interface. Additionally, the covalent bonding at the interface imparts exceptional mechanical stability, allowing the Ta/TaS_2_ catalyst to operate at a low overpotential of 398 mV for over 200 h at a current density of 2000 mA cm⁻^2^ (Figure 14g), laying the groundwork for utilizing such monolithic catalysts to promote the HER process.^[^ ^103^ ^]^ Building on this, Wang et al. employed in situ plasma techniques to modulate the active surface of MoS_2_, preparing vertically aligned mixed‐phase MoS_2_ (1T/2H‐MoS_2_@Mo). The asymmetric structure at the 1T/2H‐MoS_2_ grain boundaries leads to an uneven charge distribution on the MoS_2_ surface, thereby activating the typically inert sulfur sites. Meanwhile, the band overlap between the Mo substrate and the 1T/2H‐MoS_2_ active layer provides superior structural stability to the carrier/active layer interface, further enhancing the HER process.^[^ ^146^ ^]^ In summary, interface synergistic effects form ohmic contacts that optimize electron capture and transfer, enhance catalytic selectivity, and boost the overall durability of the system, significantly enhancing the efficiency of electrocatalytic processes. By precisely designing material combinations and interface structures, control over reaction pathways and substantial improvements in catalytic activity can be achieved, providing new strategies and pathways for efficient energy conversion (**Table** **2**).

**Table 2:** Summary of reported ohmic contact catalysts.

Ohmic contact electrocatalyst	Ohmic contact strategy	Electron transfer	Reaction	Refsf.
Cu_x_Mo_6_S_8_	Orbital hybridization and interface bonding	Cu to Cu_x_Mo_6_S_8_	Electrocatalytic hydrogen evolution	[135]
Ta/TaS_2_ MC	Interface bonding	Ta to TaS_2_	Electrocatalytic hydrogen evolution	[103]
1T/2H‐MoS_2_@Mo	Edge contact and interface bonding	Mo to 1T/2H‐MoS_2_	Electrocatalytic hydrogen evolution	[147]
Mo/MoSe_2_	Edge contact and interface bonding	Mo to MoSe_2_	Electrocatalytic hydrogen evolution	[102]
Co‐B@CoO_x_	Work function control	Co‐B to CoO_x_	Electrocatalytic nitrate reduction reaction	[147]
Pt@NiFc‐MOF	Work function control	NiFc‐MOF to Pt	Hydrazine oxidation reaction	[148]
Cd/CdS	Work function control	CdS to Cd	Photocatalytic hydrogen evolution	[149]
CMS	Orbital hybridization and interface bonding	Cu to CMS	Electrocatalytic hydrogen evolution	[95]
Co/CoP	Orbital hybridization and interface bonding	CoP to Co	Electrocatalytic water splitting	[150]
Pt/PCN‐224	Work function control	PCN‐224 to Pt	Photocatalytic oxidation of benzyl alcohol	[151]
MoC/N_x_C	Edge contact and interface bonding	NC to MoC	CO_2_ hydrogenation to formic acid	[152]
Ni/V_2_O_3_	Work function control and interface bonding	Ni to V_2_O_3_	Electrocatalytic hydrogen evolution and urea oxidation reaction	[153]
Cu/polyimide	Work function control	Cu to polyimide	Electrocatalytic nitrogen reduction	[154]
Co/N_x_C	Orbital hybridization and interface bonding	Co to NC	Oxidation of alcohols to esters	[155]
Ni/NCF	Work function control	Ni to NCF	Methanol dehydrogenation	[156]
Ru/POC	Work function control	Ru to POC	Electrocatalytic reduction of NO_x_ in water to ammonia	[157]
Fe/NC	Work function control	Fe to NC	Electrocatalytic oxygen reduction reaction	[158]
W_2_C/NC	Orbital hybridization and interface bonding	W_2_C to NC	Electrochemical alkoxylation and hydrogen evolution	[159]

^a)^ MOF: metal−organic framework;

^b)^ FCN‐224: metal−organic framework;

^c)^ Nitrogen doped carbon;

^d)^ NCF: Nitrogen‐doped carbon foam;

^e)^ P‐O doped carbon.

## 6 Conclusion and Outlook

As the third component of composite electrocatalysts consisting of metals and 2D TMDs, the interactions at the interface determine the synergistic actions of the other two components in specific electrochemical reactions. Therefore, the synthesis and regulation of this often unseen interface are critical for enhancing the activity of metal‐2D TMDs composite electrocatalysts, surpassing the performance of either the metal or the 2D TMDs alone. Ohmic contact interfaces, with efficient electron transfer pathways, fundamentally address the issue of high contact resistance caused by fermi level pinning and schottky barriers during the contact process between metals and 2D TMDs. When metals and TMDs exhibit van der Waals contact, the focus is on the difference in work functions between the metals and TMDs, which drives charge redistribution and facilitates electron transfer from the metal to the 2D TMDs. This creates electron‐enriched areas at the interface, reducing interface resistance, promoting the formation of ohmic contacts, selectively stabilizing or activating certain reactants, and accelerating specific electrocatalytic reactions. Conversely, when metals and 2D TMDs exhibit non‐van der Waals contact, the focus is on the overlapping effects between the free electrons of the metal and the valence electrons of the TMDs, leading to the formation of covalent bonds or fluctuations beyond van der Waals forces. This creates efficient pathways for electron transfer at the interface, forming ohmic contacts that enhance the electron transfer rate and stability of the catalytic process.

Although research on metal‐2D TMDs interface structures has advanced rapidly in the field of solid‐state electronics in recent years, its exploration as customizable and sustainable electrocatalysts in the electrochemical domain remains in its infancy. Therefore, to assist researchers in exploring scientific opportunities in this field, we offer some insights into this topic.

### 6.1 Optimizing the Metal‐2D TMDs Interface Structure from an Electrochemical Perspective to Construct Ohmic Contacts with High Electron Mobility

From the perspective of experimental design, it is crucial to develop advanced synthesis techniques that precisely control the morphology, composition, and interface structure of catalysts, thereby maximizing the benefits of both metals and 2D TMDs. This involves modifying the parameters of the metal or 2D TMDs components within metal‐2D TMDs electrocatalytic materials to design more efficient heterogeneous catalysts. For instance, regulating the crystal phase structure, crystallinity, and geometric morphology of the metal or 2D TMDs components can enhance interfacial synergistic effects. Furthermore, specific control over interfacial structures, such as the directed growth of high‐energy transition states or the design of high‐quality, defect‐free interfaces is essential to boost electron mobility at the interface, thereby optimizing the catalytic performance of metal‐2D TMDs electrocatalysts.

### 6.2 Developing Advanced Characterization Techniques for Visualizing the Interface Structure of Metal‐2D TMDs Electrocatalysts

As mentioned previously, the controllable interface structure between metals and 2D TMDs enables manipulation of the electronic band structure of metal‐semiconductor electrocatalysts, selectively stabilizing and/or activating specific reactants. However, the complexity of these interface structures and their nanoscale dimensions pose significant challenges in characterization. For example, traditional sample preparation processes may not preserve the integrity of the sample or capture the microscale details required. Conventional electron microscopy has limitations in revealing high‐resolution, atomic‐level details of metal‐semiconductor interfaces. Additionally, the photosensitivity of certain 2D TMDs complicates the accuracy of spectroscopic characterization. With the development of various in situ characterization methods, further mechanistic studies are necessary to fully elucidate the relationship between fermi level pinning, schottky barriers, contact resistance, and catalytic reactions, aiming to better understand the quantitative or at least semi‐quantitative structure‐performance relationships in metal‐2D TMDs electrocatalyst interface effects. Future advancements may involve improving the resolution of electron microscopes to achieve atomic‐level characterization of interface structures, enabling more detailed investigations of atomic arrangements and defects at metal‐semiconductor interfaces. Additionally, the application of artificial intelligence and machine learning techniques could lead to the development of intelligent data processing and analysis tools, facilitating the efficient handling of large‐scale data and the automated identification and analysis of interface structures.

### 6.3 Utilizing Computational Models to Accurately Simulate the Dynamics and Complexity of Metal‐2D TMDs Electrocatalysts in Reaction Systems

The application of DFT has successfully unveiled molecular‐level reaction mechanisms and active sites within catalytic systems. However, constructing appropriate metal‐2D TMDs interface structures may involve complex combinations of 2D TMDs, carriers, and inducing conditions. Moreover, in electrocatalytic reactions, the interface effects between metal carriers and semiconductor 2D TMDs on the catalyst's band position, electronic structure, structural stability, and the rate of charge transfer during reaction processes remain unclear. Developing new computational methods and constructing multiscale theoretical models are essential for accurately simulating metal‐TMDs interfaces and revealing realistic reaction pathways, thereby providing a more comprehensive understanding of catalytic mechanisms.

### 6.4 Advancing the Development of Metal‐2D TMDs Electrocatalysts in the Field of Energy Conversion

Designing metal‐2D TMDs electrocatalytic materials with specific electronic structures can significantly enhance the activation and synthesis of particular reactions by providing functional active centers. These catalysts maintain high efficiency and sustainability for targeted reactions, even in complex multicomponent reaction environments. Under appropriate stoichiometric conditions, these active sites selectively facilitate in situ water electrolysis, CO_2_ reduction, nitrogen reduction, and hydrogenation reactions, producing clean energy or high‐value products such as H_2_, O_2_, H_2_O_2_, methanol, formic acid, NH_3_, and N_2_H_4_.

## Conflict of Interest

The authors declare no conflict of interest.
